# Artificial Intelligence-Driven Reproductive Bioengineering: Integrating Fertility Diagnostics, Organ-on-Chip Systems, Cryobiology and Epigenetic Safety for Precision Reproductive Medicine

**DOI:** 10.3390/bioengineering13090976

**Published:** 2026-08-25

**Authors:** Mohamad Warda, Ali Doğan Ömür, Hae-Jin Park, Jaehoon Bae, A. M. Abd El-Aty

**Affiliations:** 1 Department of Physiology, Faculty of Veterinary Medicine, Atatürk University, 25240 Erzurum, Türkiye; 2 Department of Biochemistry and Molecular Biology, Faculty of Veterinary Medicine, Cairo University, Giza 12211, Egypt; 3 Department of Reproduction and Artificial Insemination, Faculty of Veterinary Medicine, Atatürk University, 25240 Erzurum, Türkiye; alidogan@atauni.edu.tr; 4 Department of Biochemistry, Faculty of Veterinary Medicine, Atatürk University, 25240 Erzurum, Türkiye; 5 Department of Foodcare YAKSUN, Daegu Haany University, Gyeongsan-si 38610, Republic of Korea; hjpark@dhu.ac.kr; 6 Functional Food Research Institute, Industry-University Cooperation Foundation, Daegu Haany University, Gyeongsan-si 38610, Republic of Korea; 7 Department of Food Industry, Daegu Haany University, Gyeongsan-si 38610, Republic of Korea; 8 Department of Pharmacology, Faculty of Veterinary Medicine, Cairo University, Giza 12211, Egypt; abdelaty44@hotmail.com; 9 Department of Medical Pharmacology, Faculty of Medicine, Atatürk University, 25240 Erzurum, Türkiye

**Keywords:** precision reproductive medicine, artificial intelligence in reproductive medicine, reproductive bioengineering, organ-on-chip systems, microphysiological systems, precision cryobiology, developmental epigenetics

## Abstract

Traditional assisted reproductive technologies (ART) remain constrained by subjective, descriptive diagnostics and empirical, one-size-fits-all preservation strategies that expose gametes to nonphysiological stressors, risking disruptions to cellular homeostasis and epigenetic programming. This review explores the technological convergence of artificial intelligence (AI), reproductive organ-on-chip bioengineering, multiomics, and translational cryobiology and proposes an integrated, systems-level paradigm for next-generation precision reproductive medicine. By evaluating the clinical readiness, mechanistic insights, and translational trajectories of these emerging platforms, we show how AI architectures transition fertility diagnostics from descriptive metrics to predictive computational phenotyping by integrating high-dimensional imaging, multiomics, and sperm functional datasets. Concurrently, microphysiological platforms—such as testis-, ovary-, and endometrium-on-a-chip systems—recapitulate complex multicellular architecture and endocrine dynamics. When embedded with miniaturized biosensors and machine learning loops, these “smart” closed-loop microfluidic devices enable real-time biological monitoring and adaptive culture regulation. Furthermore, integrating AI analytics into cryobiology optimizes nonlinear thermodynamic variables, shifting the field from basic postthaw morphologic survival toward safeguarding macromolecular fidelity, mitochondrial competence, and long-term epigenetic safety across lifespans and generations. Ultimately, this computational–bioengineering roadmap transitions reproductive healthcare from a reactive discipline into a predictive, personalized, and adaptive framework. Overcoming persistent challenges in biological complexity, data interoperability, and multicenter clinical validation will lead to the establishment of safe, scalable, and ethically governed healthcare infrastructures capable of protecting developmental integrity.

## 1. Introduction

Infertility affects nearly 15–20% of couples worldwide and represents a major public health challenge linked to environmental stressors, endocrine disruptors, metabolic disease, and delayed parenthood [1]. Despite advances in assisted reproductive technologies (ARTs), fertility diagnostics remain limited by subjective semen evaluation and an incomplete understanding of reproductive mechanisms. Moreover, interventions such as cryopreservation, *in vitro* culture, and intracytoplasmic sperm injection (ICSI) expose gametes and embryos to nonphysiological stressors that may induce oxidative damage, mitochondrial dysfunction, and epigenetic alterations, with potential long-term consequences for offspring health [2,3]. Moreover, reproductive medicine is undergoing a technological transformation driven by the convergence of artificial intelligence (AI), organ-on-chip bioengineering, systems biology, cryobiology, and epigenomics. AI-based approaches are being increasingly investigated for the analysis of reproductive imaging, sperm functional parameters, and molecular datasets, including metabolomic and other omics-based profiles, with the aim of developing more predictive models of reproductive function [4,5,6,7,8], whereas reproductive organ-on-chip systems provide physiologically relevant platforms for modeling reproductive tissues, spermatogenesis, endocrine signaling, and reproductive toxicology [9,10,11].

Advances in cryobiological engineering—including intelligent vitrification, nanowarming, and digitally integrated cryobanking—are improving fertility preservation and tissue cryostorage strategies [12,13,14]. Although assisted reproductive technologies (ARTs) have transformed the management of infertility over the past four decades, clinical decision-making remains largely dependent on conventional diagnostic paradigms that provide limited insight into the complex biological processes governing reproductive competence.

In male infertility, routine semen analysis continues to rely primarily on the sperm concentration, motility, and morphology, which are parameters that often correlate poorly with the fertilization potential, embryo development, and reproductive outcomes [15]. Fertility is increasingly understood as a systems-level process shaped by interactions among genetic, epigenetic, metabolic, endocrine, and environmental factors, including mitochondrial activity, oxidative stress, chromatin organization, DNA integrity, noncoding RNA cargo, cellular signaling, and the reproductive microenvironment.

Concerns are also growing regarding the biological consequences of reproductive interventions themselves. In human clinical reports, procedures such as sperm processing, cryopreservation, *in vitro* culture, and intracytoplasmic sperm injection (ICSI) expose gametes and embryos to nonphysiological conditions that have been associated with oxidative stress, membrane damage, mitochondrial dysfunction, and genomic instability, whereas animal and *in vitro* models provide mechanistic support for these observations [16,17]. Emerging evidence indicates associations between environmental and procedural stressors in ART and alterations in epigenetic programming during critical developmental windows; however, these findings remain correlational, and causality has not been established [2].

Concurrently, these technological advances are becoming increasingly integrated across reproductive research and medicine. AI-based platforms can integrate imaging, molecular, and functional datasets to generate predictive models of reproductive performance, while reproductive organ-on-a-chip systems provide physiologically relevant models of spermatogenesis, endocrine signaling, reproductive toxicology, and fertility preservation [8,11]. Advances in cryobiological engineering are similarly improving fertility preservation and tissue cryostorage strategies while addressing cellular and molecular injury [18].

Together, these innovations support a precision reproductive medicine paradigm in which diagnosis, intervention, preservation, and long-term safety assessment are integrated within biologically informed, data-driven frameworks [9,11,19]. Rather than focusing solely on fertilization success, future reproductive technologies aim to enable continuous monitoring of reproductive health, personalized treatment optimization, and predictive risk assessment across individualized reproductive trajectories [11,14]. Advances in reproductive bioengineering and epigenetic research further emphasize the importance of safeguarding developmental integrity and minimizing long-term risks across generations [13,20].

**Figure 1:**
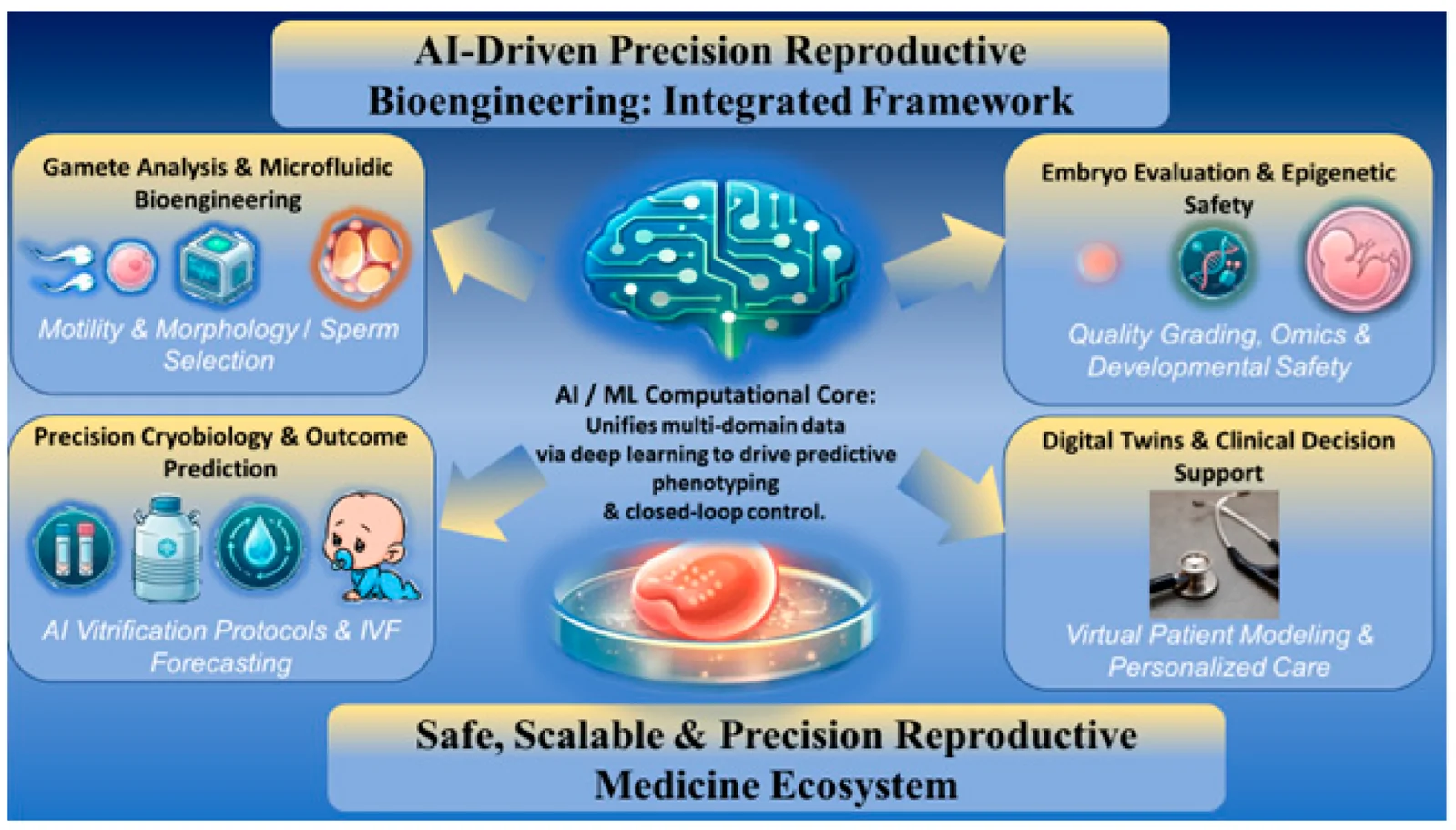
Central integrated framework for AI-driven precision reproductive bioengineering.

To address these multifaceted challenges, we propose a centralized systems framework that connects these computational, bioengineering, and biological advances (Figure 1). In this paradigm, an artificial intelligence computational engine processes multimodal datasets to coordinate four interconnected operational pillars: real-time microfluidic sperm selection and device regulation, automated embryo multiomics and epigenetic safety tracking, AI-optimized precision cryopreservation, and multiscale digital reproductive twins for personalized clinical decision support.

The central artificial intelligence and machine learning (AI/ML) computational core functions as an integrative engine, processing multidomain biological data through deep learning architectures to drive predictive computational phenotyping and closed-loop system control. This centralized core coordinates four interconnected operational pillars: gamete analysis and microfluidic bioengineering for automated sperm selection and real-time microfluidic device regulation; embryo evaluation and epigenetic safety focusing on morphological quality grading, multiomics profiling, and long-term developmental safety tracking; precision cryobiology and outcome prediction for AI-optimized vitrification protocols and pregnancy forecasting; and digital reproductive twins for multiscale virtual patient modeling and personalized clinical decision support to deliver a safe, scalable, and precise reproductive medicine ecosystem.

In this context, a synthesis that examines these technological domains not only individually but also in relation to their potential convergence and translational implementation is needed. This Review examines the convergence of these technologies and proposes an integrated translational framework combining AI-driven analysis, engineered reproductive systems, optimized cryopreservation strategies and epigenetic risk evaluation while outlining a forward-looking roadmap and the scientific, regulatory and ethical challenges relevant to clinical and industrial translation.

### Novelties and Contributions

This review provides an integrated and multidisciplinary perspective on emerging technologies in precision reproductive medicine, combining artificial intelligence, reproductive bioengineering, reproductive microphysiological systems, precision cryobiology, multiomics, digital reproductive twins, and developmental and epigenetic safety within a unified translational framework. Its main contributions are fourfold. First, it connects AI-based fertility diagnostics and multimodal reproductive phenotyping with engineered reproductive models rather than considering these technologies as isolated developments. Second, it links advances in reproductive organ-on-chip systems and computational modeling with precision cryobiology and fertility preservation, emphasizing their potential integration into a continuous reproductive-care ecosystem. Third, the review explicitly incorporates developmental, genomic, and epigenetic integrity as essential dimensions of technological evaluation, extending assessment beyond conventional procedural or fertilization outcomes. Fourth, it proposes a technology maturity framework and a 2026–2045 technology roadmap that distinguishes established and experimentally demonstrated applications from emerging and conceptual technologies and future projections. Collectively, these contributions provide a translational framework for evaluating how biological, computational, and engineering innovations may converge while identifying the validation, safety, infrastructural, ethical, and regulatory requirements necessary for responsible clinical translation.

Recent reviews have addressed important individual components of emerging reproductive technologies, including artificial intelligence in assisted reproductive technology [21], reproductive organ-on-chip systems [22,23], epigenetic alterations associated with ART [24], digital twins in fertility and ART [25], AI-assisted cryopreservation [26], and the integration of multiomics and artificial intelligence for preserving ovarian tissue fertility [8]. These studies provide important domain-specific foundations; however, the present review differs in its systems-level integration of these technological domains within a unified precision reproductive-medicine framework, together with explicit consideration of technological maturity, translational readiness, developmental and epigenetic safety, regulatory requirements, and future technology trajectories.

To clarify the scope and distinct contribution of the present review, Table 1 compares the major technological domains addressed in recent reviews with those integrated in the present work. The comparison highlights that although individual domains, such as AI, organ-on-chip systems, epigenetics, cryopreservation, multiomics, and digital twins have been reviewed separately, the present review considers these technologies within a unified translational framework that explicitly incorporates technological maturity, developmental and epigenetic safety, validation requirements, regulatory considerations, and projected trajectories.

## 2. Review Methodology

### 2.1. Review Design and Scope

This article presents a structured integrative review of emerging evidence at the intersection of AI, reproductive bioengineering, fertility diagnostics, reproductive microphysiological systems, cryobiology, multiomics, digital reproductive twins, and developmental and epigenetic safety. This review aimed to integrate evidence across these rapidly developing fields and examine their potential convergence within a precise reproductive-medicine framework. The review focuses on four major questions: (i) the application of AI and multimodal data analysis to fertility diagnosis and prediction; (ii) the development of organ-on-chip and other reproductive microphysiological systems for modeling reproductive physiology, disease, toxicology, and fertility preservation; (iii) the integration of computational approaches and advanced bioengineering into reproductive cryobiology; and (iv) the incorporation of developmental, genomic, and epigenetic safety into the evaluation of emerging reproductive technologies.

### 2.2. Literature Search and Selection

A structured literature search was performed using PubMed/MEDLINE, Web of Science, Scopus, and EMBASE, covering publications from 2004 to 2026. Search terms were developed around the primary domains of the review and combined using Boolean operators (AND/OR). The main search terms included “artificial intelligence,” “machine learning,” “deep learning,” “fertility,” “infertility,” “assisted reproductive technology,” “sperm,” “oocyte,” “embryo,” “reproductive medicine,” “organ-on-chip,” “microphysiological system,” “microfluidics,” “organoid,” “tissue engineering,” “cryopreservation,” “vitrification,” “cryobiology,” “cryobanking,” “fertility preservation,” “multiomics,” “epigenomics,” “DNA methylation,” “chromatin,” “developmental programming,” “epigenetic safety,” “digital twin,” and “predictive modeling.” Related synonyms and controlled vocabulary were also used where applicable to each database. Studies were considered relevant when they provided experimental, clinical, computational, mechanistic, or translational evidence addressing at least one of the major themes of the review. Priority was given to human reproductive studies, human-derived models, clinically relevant reproductive technologies, and translational bioengineering applications, while relevant animal and *in vitro* studies were included when important mechanistic or translational evidence was provided. Original research articles, clinical studies, methodological papers, and relevant high-quality reviews were considered. Studies unrelated to reproductive biology or technology or lacking sufficient relevance to the objectives of the review were excluded.

### 2.3. Inclusion and Exclusion Criteria

Studies were included when they addressed at least one of the principal themes of the review and provided relevant experimental, clinical, computational, mechanistic, or translational evidence. Priority was given to studies involving human reproductive biology, human-derived reproductive models, clinically relevant assisted reproductive technologies, and translational reproductive bioengineering. Relevant animal and *in vitro* studies were also included when important mechanistic or translational evidence was provided. Eligible publication types included original research articles, clinical studies, methodological studies, and relevant high-quality reviews. Studies were excluded if they were unrelated to reproductive biology or reproductive technologies, focused exclusively on nonreproductive applications without clear relevance to the objectives of the review, or did not provide sufficient scientific or methodological information to support the topics addressed. Publications without substantive relevance to AI, reproductive bioengineering, fertility, cryobiology, reproductive microphysiological systems, multiomics, developmental biology, or epigenetic safety were also excluded.

### 2.4. Data Synthesis

The relevant literature was organized thematically according to the major technological and biological domains addressed in the manuscript, including AI-assisted fertility diagnostics, reproductive digital twins, reproductive organ-on-chip systems, AI-integrated microphysiological platforms, precision cryobiology, nanowarming and smart cryobanking, developmental and epigenetic integrity, and regulatory and ethical considerations. The evidence was synthesized qualitatively, with emphasis on biological relevance, technological maturity, demonstrated performance, translational readiness, and reported limitations. Technology maturity was classified using predefined qualitative criteria: “clinical” referred to technologies with demonstrated clinical use or implementation in reproductive practice; “translational” referred to technologies supported by substantial experimental evidence and validation in clinically relevant models with a plausible pathway toward clinical application; “experimental” referred to technologies demonstrated primarily in laboratory, animal, or *in vitro* systems without established clinical validation; “emerging/conceptual” referred to approaches supported by early proof-of-concept evidence or proposed integration of existing technologies but lacking sufficient validation for clinical translation; and “conceptual” referred to forward-looking approaches for which clinical feasibility remains largely hypothetical. These categories were used to distinguish technological maturity rather than to represent formal regulatory approval or standardized regulatory technology readiness levels. Particular attention was given to distinguishing established or experimentally demonstrated applications from emerging and conceptual technologies.

Because the reviewed technologies span substantially different stages of development, their clinical potential was considered in relation to validation, reproducibility, model relevance, dataset limitations, external validation, standardization, and regulatory requirements. This approach was selected because the purpose of the review was to provide an integrated, multidisciplinary synthesis of rapidly evolving technologies rather than a quantitative meta-analysis of comparable interventions or outcomes. The resulting framework therefore emphasizes both current evidence and translational opportunities while recognizing the limitations and uncertainties associated with emerging reproductive technologies.

## 3. AI-Assisted Fertility Diagnostics

The growing recognition of infertility as a complex, multifactorial condition has exposed the limitations of conventional diagnostic approaches that rely primarily on isolated laboratory measurements [1]. Although standard semen analysis remains the cornerstone of male fertility assessment, its ability to predict reproductive outcomes is often inconsistent, reflecting the intricate biological processes that underlie fertilization, embryo development, and successful pregnancy [27]. Advances in molecular biology have revealed that reproductive competence is influenced by a broad spectrum of factors, including genomic integrity, mitochondrial function, oxidative stress, epigenetic regulation, and interactions within the reproductive microenvironment [19,28]. The increasing availability of high-dimensional biological data, coupled with rapid progress in computational sciences, has therefore created a strong rationale for the application of AI in reproductive medicine [29].

AI technologies are uniquely positioned to transform fertility diagnostics by integrating heterogeneous datasets that would be difficult to interpret using conventional analytical approaches [19,30]. Through machine learning and deep learning algorithms, AI systems can identify complex patterns across imaging, molecular, functional, and clinical data, enabling more comprehensive assessments of reproductive health [19,31]. Beyond improving diagnostic accuracy, these approaches facilitate a transition toward predictive and personalized fertility medicine, in which reproductive outcomes can be estimated, treatment strategies optimized, and biological risks identified before clinical intervention [32,33]. As a result, AI is increasingly viewed not only as an analytical tool but also as a foundational technology for the next generation of precision reproductive medicine [29].

### 3.1. Transition from Descriptive to Predictive Fertility Medicine

Traditional fertility assessment has historically relied on manual or semiautomated semen analysis, focusing primarily on sperm concentration, motility, and morphology. While these parameters provide valuable baseline information, they frequently fail to capture the biological complexity that determines fertilization potential and subsequent embryo competence [15]. Furthermore, conventional assessments often exhibit substantial interobserver variability and limited predictive power when applied to individual clinical outcomes.

AI is beginning to reshape this paradigm by enabling computational fertility phenotyping on the basis of multidimensional biological datasets. Deep learning architectures, including convolutional neural networks (CNNs), transformer-based image analysis systems, and advanced machine learning algorithms, have demonstrated considerable potential for automated sperm classification, motility tracking, morphological assessment, and prediction of fertilization outcomes [19,31]. These systems can process large volumes of data with high consistency, reducing subjectivity while uncovering biologically relevant patterns that may not be apparent through conventional analysis.

Importantly, contemporary AI-driven fertility platforms extend far beyond microscopy image interpretation. Contemporary AI-driven fertility research is also expanding beyond conventional microscopy-based assessments toward computational analysis and the potential integration of information derived from computer-assisted sperm analysis (CASA), Raman spectroscopy, metabolomics, proteomics, transcriptomics, oxidative stress biomarkers, and sperm DNA fragmentation testing. These complementary modalities capture molecular, cellular, and physiological dimensions of reproductive function, including DNA integrity, metabolic activity, and oxidative damage signatures [6,34].

As these technologies mature, they may enable individualized fertility probability scores, predictive treatment–response models, and digital reproductive profiles that support precision-guided clinical decision-making, particularly through AI-based integration of sperm function tests and genomic integrity markers [35]. Ultimately, the convergence of AI, multiomics, and advanced reproductive analytics may shift fertility medicine from a predominantly descriptive discipline toward a predictive and data-driven model in which sperm selection and treatment planning are guided by integrated molecular–computational signatures rather than conventional semen parameters alone [7]. However, these prospective possibilities should be distinguished from computational approaches that have already undergone quantitative validation.

While early applications of machine learning in reproductive medicine focused predominantly on exploratory image analysis and feature extraction, an increasing number of validation studies now provide quantitative evidence that computational approaches can perform specific reproductive assessment tasks, although clinical utility remains heterogeneous and incompletely established. For example, deep-learning analysis has been applied to the quantitative classification of human sperm motility patterns, demonstrating the feasibility of automated computational assessment of sperm movement characteristics [36]. With respect to embryo assessment, Bormann et al. (2020) [19] evaluated deep-learning models using images from 742 embryos from 97 patients and reported 90% accuracy in selecting the highest-quality embryo; a separate model trained to assess implantation potential using 97 euploid embryos achieved 75.26% performance, compared with 67.35% among 15 trained embryologists (*p* < 0.0001). Similarly, Khosravi et al. (2019) [37] reported an AUC of 0.987 for deep-learning classification of blastocyst quality on a blind test set; importantly, this metric reflects prediction of embryo-quality classification rather than direct prediction of pregnancy or live birth. At the clinical level, prospective evidence has begun to test whether computational performance translates into patient benefit. In a multicenter, randomized, double-blind, noninferiority trial involving 1066 patients, deep learning-assisted embryo selection resulted in a clinical pregnancy rate of 46.5% (248/533), compared with 48.2% (257/533) with conventional morphology-based selection (risk difference, −1.7 percentage points; 95% CI, −7.7 to 4.3; *p* = 0.62), and noninferiority of deep learning was not demonstrated. Live birth occurred in 39.8% of patients in the deep learning group compared with 43.5% in the conventional morphology group (risk difference, −3.9 percentage points; 95% CI, −9.9 to 2.2; *p* = 0.24). These findings provide important clinical evidence that quantitative algorithmic performance does not necessarily translate into improved reproductive outcomes and underscore the need for prospective outcome-based validation before AI-assisted embryo selection can be considered clinically superior to conventional assessment [29]. Collectively, these findings indicate that the transition from descriptive computational applications toward quantitatively evaluated predictive approaches is underway, although the extent of clinical translation remains limited and heterogeneous. Building on these individually evaluated approaches, the present review proposes the integration of multiomic profiling, real-time microfluidic sensing, cryobiological monitoring, and predictive machine learning into a potentially closed-loop assisted reproductive framework; however, this integrated architecture remains a proposed research framework rather than a clinically validated system and requires prospective evaluation of its accuracy, safety, clinical benefit, interoperability, and cost-effectiveness.

Experimental *in vitro* spermatogenesis should also be interpreted according to species-specific evidence. Complete spermatogenesis resulting in functional sperm capable of producing offspring has been demonstrated in mouse models [38,39]. However, human *in vitro* spermatogenesis has not yet been shown to result in the production of fully functional haploid male gametes with demonstrated reproductive competence. Current human approaches have generally achieved incomplete differentiation, and substantial technical and ethical barriers remain before clinical application can be considered [38]. Accordingly, testis-on-chip and related reproductive biotechnologies should currently be regarded as experimental platforms, with the available evidence derived predominantly from animal models, including mouse and porcine systems, rather than from established human clinical applications.

### 3.2. AI and Sperm Functional Integrity

The assessment of sperm functional integrity has emerged as a critical component of modern fertility evaluation, extending beyond conventional measures of concentration, motility, and morphology. Among the most extensively studied biomarkers, sperm DNA fragmentation (SDF) is associated with impaired fertilization, reduced embryo quality, recurrent pregnancy loss, and lower success rates following assisted reproductive technology (ART), particularly in clinical settings such as recurrent implantation failure and unexplained infertility [40].

Recent evidence further reinforces the prognostic relevance of SDF for both natural conception and ART outcomes, highlighting its role as an independent indicator of oxidative stress–mediated sperm damage and compromised genomic integrity [1,41].

Nevertheless, the routine clinical application of SDF testing remains constrained by methodological variability, differences in assay platforms (TUNEL, SCSA, Comet, and SCD), limited interlaboratory standardization, and challenges in integrating results into personalized reproductive decision-making frameworks [42].

Recent advances in AI offer opportunities to enhance the evaluation of sperm functional competence by integrating diverse biological and phenotypic datasets into unified predictive frameworks [30]. Rather than relying on a single biomarker, machine learning algorithms can simultaneously analyze information derived from sperm chromatin structure, DNA fragmentation indices, mitochondrial membrane potential, oxidative stress markers, metabolic activity, and high-resolution motility dynamics [43]. Such multidimensional approaches are particularly attractive because sperm dysfunction often arises from interconnected molecular pathways involving oxidative damage, mitochondrial impairment, defective chromatin packaging, and altered cellular signaling [44].

AI-assisted image analysis has also demonstrated potential for extracting previously unrecognized features from sperm morphology and movement patterns that may correlate with functional competence [45]. By combining advanced imaging with computational pattern recognition, these systems can identify subtle phenotypic signatures associated with fertilization capacity, embryo developmental potential, and reproductive outcomes [46,47]. Emerging studies further suggest that integrating molecular biomarkers with time-resolved motility trajectories may improve the prediction of sperm quality beyond the capabilities of traditional semen analysis alone [36].

From a clinical perspective, these developments support a transition from descriptive assessment toward functional fertility phenotyping. Future AI-enabled diagnostic platforms may facilitate more accurate patient stratification, identification of clinically relevant subfertile phenotypes, optimization of ART selection, and individualized treatment planning. Ultimately, the integration of functional biomarkers, advanced imaging, and machine learning analytics may provide a more comprehensive understanding of male reproductive competence and enable precision-based approaches to fertility management [48].

### 3.3. Reproductive Digital Twins

Among the emerging applications of AI in healthcare, the concept of the digital twin has attracted considerable attention as a framework for predictive and personalized medicine. A digital twin is a dynamic computational representation of an individual who continuously integrates biological, clinical, environmental, and behavioral data to model physiological processes and predict future outcomes [49]. While digital twin technologies have gained traction in cardiovascular medicine, oncology, and metabolic disease management, their application to reproductive medicine remains in its early stages but holds significant transformative potential [25].

The biological complexity of human reproduction makes it particularly well suited to digital twin approaches. Reproductive outcomes are influenced by the interplay of numerous factors, including endocrine regulation, genetic and epigenetic variation, gamete quality, environmental exposure, age-related physiological changes, and lifestyle determinants. Conventional fertility assessments often capture only a limited snapshot of this complexity, making it difficult to predict treatment outcomes at the individual level. Digital twin systems seek to address this limitation by integrating multidimensional datasets into continuously updated computational models capable of simulating reproductive function and forecasting clinically relevant outcomes [48,49].

In the context of male fertility, future reproductive digital twins could incorporate endocrine profiles, genomic and epigenomic information, sperm functional characteristics, oxidative stress biomarkers, metabolomic signatures, environmental exposures, microbiome composition, and longitudinal reproductive histories. By combining these data streams with machine learning algorithms and mechanistic biological models, digital twins may provide individualized representations of reproductive health that evolve over time in response to physiological and environmental changes [48,49,50].

Such systems could eventually support a wide range of clinical applications. For example, digital twins may help estimate the likelihood of successful sperm cryopreservation by modeling susceptibility to oxidative stress, membrane injury, and postthaw functional decline. Integration of gamete characteristics with embryological and clinical data may also improve the prediction of embryo developmental competence and treatment outcomes following assisted reproductive technologies. Furthermore, longitudinal modeling of hormonal dynamics, molecular biomarkers, and environmental influences could facilitate personalized assessment of reproductive aging trajectories and identify individuals at increased risk of fertility decline before clinically evident dysfunction occurs [48,49,51].

Beyond treatment optimization, digital twins may provide a framework for evaluating the long-term biological consequences of reproductive interventions. Integration of epigenetic, developmental, and clinical outcome data could enable estimation of intervention-associated risks and support the design of safer, individualized reproductive strategies. Although substantial technical, computational, and regulatory challenges remain, the convergence of artificial intelligence, multiomics technologies, advanced reproductive diagnostics, and longitudinal health data is creating the foundation for future digital twin ecosystems [20,52,53].

Ultimately, reproductive digital twins represent a conceptual shift from episodic fertility assessment toward continuous, data-driven reproductive health management. By enabling individualized prediction, simulation, and optimization of reproductive outcomes, these technologies may help transform reproductive medicine from a predominantly reactive discipline into a predictive and precision-oriented field [52].

Collectively, these developments demonstrate that AI is rapidly evolving from being a supportive analytical tool to being a foundational component of precision reproductive medicine. Current applications range from automated fertility diagnostics and functional sperm assessment to predictive reproductive modeling and digital twin technologies. The major AI-driven innovations, their current development status, their principal advantages, and their remaining limitations are summarized in Table 2.

### 3.4. Explainable Artificial Intelligence and Algorithmic Transparency in Reproductive Medicine

Despite the high predictive performance of deep learning (DL) architectures in automated sperm tracking, sperm morphological assessment, embryo grading, and blastocyst selection, their clinical translation remains constrained by the limited interpretability of many complex models. In assisted reproductive technology (ART), where AI-generated predictions may influence sperm selection, embryo prioritization, and treatment decisions, predictive accuracy alone may be insufficient to establish clinical utility. Clinicians and embryologists require sufficient insight into the factors underlying an algorithmic prediction to assess its biological plausibility, recognize potential errors or biases, and determine whether the model is appropriate for clinical implementation. Recent reviews of AI in ART have consequently highlighted interpretability and transparency as important considerations in the transition from high-performing research models to clinically deployable decision-support systems [5,54,55]. In this context, explainable artificial intelligence (XAI) has emerged as an important framework for improving the transparency and auditability of AI-assisted reproductive decision-making, particularly through feature-attribution and image-based explanation methods such as SHAP, LIME, Grad-CAM, and related saliency approaches [55]. However, explainability should not be considered a substitute for rigorous external validation, calibration, prospective evaluation, or demonstrated clinical benefit. Rather, XAI should complement these requirements by helping clinicians and researchers understand model behavior, identify potentially inappropriate decision patterns, and critically evaluate whether algorithmic predictions are consistent with clinically and biologically meaningful information [55].

XAI approaches can broadly be divided into intrinsically interpretable models and post hoc explanation methods. Intrinsically interpretable approaches incorporate transparency directly into the model architecture, whereas post hoc methods seek to explain the predictions of otherwise complex models after they have been trained. The latter are particularly relevant to deep neural networks used in reproductive imaging because these models can achieve high predictive performance while providing limited information about the biological features responsible for their decisions [54,55].

For image-based applications, including automated sperm analysis and time-lapse embryo assessment, visual attribution methods such as gradient-weighted class activation mapping (Grad-CAM), integrated gradients, and saliency mapping can provide spatial explanations of model predictions [54,56,57]. Grad-CAM uses gradients associated with the predicted class to identify image regions that contribute most strongly to a model’s decision, thereby generating heatmaps that can be superimposed on the original image predictions [54,56]. Integrated gradients attribute the prediction to individual input pixels by integrating model gradients along a defined path between a baseline input and the observed image, whereas saliency methods estimate the sensitivity of the prediction to changes in individual image feature predictions [54]. In reproductive applications, these approaches can help determine whether a model focuses on biologically meaningful structures, such as sperm head morphology, acrosomal integrity, midpiece characteristics, flagellar features, or embryo structures, including the inner cell mass and trophectoderm, rather than irrelevant imaging artifacts, background regions, or differences in image acquisition [54,56,57,58].

For multimodal models incorporating clinical characteristics, sperm functional assays, imaging-derived variables, and multiomics data, feature-based attribution methods such as SHAP (SHapley Additive exPlanations) and LIME (Local Interpretable Model-agnostic Explanations) provide complementary forms of interpretability acquisition [54,58]. SHAP estimates the contribution of individual features to a specific prediction based on the Shapley-value framework, allowing researchers to determine whether variables such as sperm DNA fragmentation, oxidative stress, mitochondrial function, maternal age, or other clinical parameters contribute positively or negatively to a predicted reproductive outcome [54,58]. SHAP can also be aggregated across a cohort to provide a broader assessment of feature importance and model behavior. Recent multicenter work in assisted conception has demonstrated the practical utility of explainable AI and SHAP for identifying clinically informative predictors across large IVF datasets, including follicular characteristics associated with oocyte yield and subsequent reproductive outcomes [5]. In contrast, LIMEs construct a locally interpretable surrogate model around an individual prediction and can therefore be used to identify the variables that most strongly influence a particular patient-level or sample-level decision [54,58]. These methods are especially relevant to precision reproductive medicine because they can provide individualized explanations rather than relying exclusively on population-level measures of feature importance. However, feature-attribution results should be interpreted cautiously, particularly when predictors are correlated or derived from observational clinical data, because an important model feature does not necessarily represent a causal determinant of the reproductive outcome [54,59].

Importantly, different XAI approaches provide different types of explanations and should therefore not be regarded interchangeably. Grad-CAM and related attribution methods are particularly suitable for identifying where an image-based model is obtaining predictive information, whereas SHAP and LIME are more appropriate for examining which clinical, physiological, or molecular variables contribute to a prediction [54,57,58]. Furthermore, concept-based approaches attempt to move beyond low-level feature attribution by relating model representations to predefined or biologically interpretable concepts [60]. In reproductive medicine, such approaches could help determine whether latent representations learned by a neural network correspond to clinically meaningful concepts, such as sperm motility patterns, chromatin integrity, embryo developmental morphology, or cellular organization. Recent work in embryo assessment has demonstrated the feasibility of combining latent-space representations with visual attribution and feature-based methods, such as Grad-CAM, SHAP, and LIME, to investigate the biological features underlying model predictions and to obtain expert feedback on their interpretability [60]. Integrating such concepts with established biological knowledge may therefore improve the interpretability and clinical relevance of AI-generated predictions, although the biological validity and stability of these explanations require independent validation by domain experts and across datasets [54,57,60].

Nevertheless, XAI methods have important technical, methodological, and translational limitations that should be considered before their outputs are used as evidence of biological validity or clinical reliability. Post hoc explanations do not necessarily demonstrate that a model has identified a causal biological mechanism. For example, visually plausible saliency maps may be sensitive to image noise, perturbations, model architecture, or image acquisition conditions and may therefore reflect confounding features rather than biologically meaningful determinants [54,55,58]. Similarly, SHAP and LIME explanations may vary according to feature correlations, baseline selection, perturbation strategies, and other methodological assumptions, but their application to high-dimensional datasets may also involve substantial computational demands [54,55]. In addition, seemingly intuitive explanations may create a false sense of confidence and contribute to automation bias or inappropriate reliance on AI-generated recommendations, highlighting the importance of appropriate human oversight in clinical decision-making [54,55]. Therefore, XAI outputs should not be interpreted independently of model performance, external validation, robustness testing, reproducibility, or domain-expert assessment [54,55,58]. Reproducibility of explanations across datasets, imaging platforms, clinical populations, and model architectures is particularly important in ART, where systematic differences in patient characteristics, laboratory protocols, or imaging conditions may introduce hidden sources of algorithmic bias [54,58]. Thus, although XAI can enhance transparency, auditability, and clinical communication, its outputs should be regarded primarily as descriptions of model behavior rather than as proof of causal biological relationships or clinical effectiveness [54,55].

The clinical value of XAI therefore extends beyond simply making AI predictions visually understandable. Transparent explanations can facilitate human–AI interactions, allowing embryologists and clinicians to critically evaluate algorithmic recommendations, identify potentially erroneous predictions, and determine when an AI output should be rejected or subjected to further review [54,57,61]. XAI may also support model auditing by revealing whether predictions depend disproportionately on demographic, laboratory specific, or technically induced feature reviews [54,57]. In this context, explainability can contribute to the broader requirements of trustworthy medical AI, including transparency, accountability, reproducibility, fairness, and traceability review [54]. However, explainability should be considered a complementary component of trustworthy AI rather than a substitute for external validation, bias assessment, prospective evaluation, human oversight, and appropriate clinical governance [54,57,61]. Overall, explainable AI provides an important bridge between high-performing computational models and clinically interpretable decision support in reproductive medicine. The most appropriate strategy is unlikely to depend on a single XAI method; rather, complementary approaches combining image attribution, feature-level explanations, concept-based interpretation, and biological validation may provide a more comprehensive assessment of model behavior. Future reproductive AI systems should therefore be evaluated not only on the basis of their predictive accuracy but also on the consistency, biological plausibility, robustness, and clinical usefulness of their explanations. Such a framework would strengthen clinician confidence, facilitate algorithmic auditing, and support the responsible translation of AI-based reproductive technologies into clinical practice.

### 3.5. Data Bias, Class Imbalance, and Model Generalizability

Despite the increasing use of AI in reproductive medicine, the reliability and clinical applicability of these models strongly depend on the quality, representativeness, and distribution of the datasets used for training and validation. Reproductive AI datasets may be affected by demographic underrepresentation, single-center sampling, and differences in patient characteristics, laboratory protocols, imaging systems, and clinical practices. Such variation can introduce dataset bias and may cause models to learn center-specific or acquisition-related features rather than biologically meaningful predictors. Previous work on fertility AI has emphasized that models developed from single-clinic datasets require validation in independent cohorts and that greater diversity across clinical and patient populations is important for improving generalizability [55,62].

Class imbalance represents an additional methodological challenge, particularly when models predict relatively uncommon reproductive outcomes. Imbalanced datasets can produce strong overall performance while providing inadequate prediction of minority classes, potentially resulting in biased or unreliable clinical predictions. Assisted-reproduction datasets have specifically demonstrated the importance of considering the degree of class imbalance and sample size when developing and evaluating predictive models [63]. Accordingly, performance assessment should extend beyond overall accuracy and consider sensitivity, specificity, F1 score, precision–recall performance, and calibration where appropriate. Strategies such as stratified sampling, class weighting, and controlled resampling may help mitigate imbalance but should be implemented carefully to avoid data leakage or distortion of the underlying data distribution [63]. Importantly, high performance on an internal test set does not establish clinical generalizability. External validation using independent cohorts, preferably from multiple centers and different patient populations, is needed to determine whether model performance is maintained under changes in patient characteristics, outcome prevalence, laboratory procedures, and imaging platforms. Recent multicenter validation of an AI model for predicting euploid blastocyst development from mature oocytes illustrates the value of testing models across geographically distinct clinics and patient populations before wider clinical application [64]. Prospective, multicenter validation should therefore be considered an essential step before widespread clinical implementation, allowing assessment of model performance under real-world clinical conditions, temporal changes in patient populations, and interinstitutional variability.

## 4. Testis-on-Chip and Reproductive Organ Engineering

The development of advanced reproductive technologies increasingly depends on experimental models that accurately recapitulate human physiology while providing sufficient control for mechanistic investigation. Although animal models have contributed substantially to our understanding of reproductive biology, important interspecies differences in endocrine regulation, germ-cell development, reproductive toxicology, and tissue architecture often limit their ability to predict human reproductive health and disease [10]. Similarly, conventional two-dimensional cell culture systems fail to reproduce the complex multicellular interactions and dynamic microenvironmental conditions that govern reproductive function *in vivo*. These limitations have driven rapid progress in the development of physiologically relevant experimental platforms, particularly organ-on-a-chip systems [65] and three-dimensional organoid models [66,67], which better mimic the tissue-specific architecture, fluid dynamics, and cell–cell signaling observed in human reproductive tissues. As a result, these next-generation models are being increasingly recognized as promising translational bridges between basic reproductive biology and clinical application.

Advances in tissue engineering, microfluidics, biomaterials science, and stem cell biology have led to the emergence of reproductive microphysiological systems, which are commonly referred to as organ-on-a-chip technologies [65]. These platforms are designed to recreate key structural and functional features of native tissues within controlled microengineered environments. By integrating living cells with microfluidic architectures, organ-on-a-chip systems can reproduce critical aspects of tissue organization, vascular transport, biochemical signaling, mechanical stimulation, and cell–cell communication that are difficult to achieve using conventional *in vitro* models [66]. As a result, these technologies are increasingly recognized as promising tools for studying reproductive physiology, disease mechanisms, toxicological responses, and therapeutic interventions under conditions that more closely resemble human biology [67].

### 4.1. Emergence of Reproductive Microphysiological Systems

Among reproductive organ-on-a-chip platforms, testes-on-a-chip systems have emerged as particularly promising because of the extraordinary complexity of spermatogenesis [68,69,70]. Unlike many biological processes that can be studied under relatively simplified culture conditions, sperm production depends on a highly specialized microenvironment involving coordinated interactions between germ cells, Sertoli cells, Leydig cells, peritubular myoid cells, immune components, and endocrine signaling networks. The maintenance of spermatogenesis requires precise spatial organization within the seminiferous tubules, tightly regulated hormonal gradients, appropriate oxygen and nutrient delivery, and preservation of the blood–testis barrier, which serves essential functions in germ cell protection and maturation [69,71].

Replicating these physiological conditions *in vitro* has historically been a major challenge. Testis-on-chip technologies address this challenge by combining microfluidic control with engineered tissue architectures that more closely mimic the native testicular niche [72]. Through the generation of controlled biochemical gradients, dynamic perfusion systems, and physiologically relevant cellular interactions, these platforms provide opportunities to investigate spermatogenic regulation, reproductive toxicology, fertility preservation, and testicular pathophysiology with unprecedented precision [23,72]. Consequently, testis-on-a-chip systems are increasingly viewed as critical components of next-generation reproductive bioengineering and as potential bridges between experimental reproductive science and personalized reproductive medicine [23,73].

### 4.2. Engineering Spermatogenesis In Vitro

One of the most ambitious goals of reproductive bioengineering is the recreation of functional spermatogenesis outside the human body [70,74]. Achieving this objective is particularly challenging because sperm production is among the most spatially and physiologically complex developmental processes in mammalian biology [38,75]. In the testis, germ-cell proliferation, meiosis, differentiation, and maturation occur within a highly organized microenvironment that is continuously regulated by endocrine signals, metabolic interactions, and cellular communication networks [38,75]. Consequently, successful *in vitro* spermatogenesis requires far more than simple cell culture; it necessitates reconstruction of the dynamic testicular niche that supports germ-cell development throughout multiple stages of maturation [70,74].

A major focus of contemporary reproductive engineering is the recreation of seminiferous tubule architecture, which serves as the functional unit of sperm production [38,74]. *In vivo*, the seminiferous tubules provide a precisely organized environment in which developing germ cells interact closely with Sertoli cells while remaining protected by the blood–testis barrier. Testis-on-chip platforms seek to mimic these structural relationships by spatially organizing multiple cell populations within microengineered environments that preserve physiological cell–cell communication and tissue polarity [23,72].

Particular attention has been given to reproducing the supportive functions of Sertoli cells, often referred to as the “nurse cells” of spermatogenesis [38]. Sertoli cells regulate nutrient transport, germ-cell differentiation, hormonal responsiveness, and maintenance of the blood–testis barrier, thereby providing the specialized microenvironment required for spermatogenesis [74,76]. Similarly, successful models must incorporate Leydig cell function to reproduce local testosterone production and endocrine signaling, both of which are essential for normal germ-cell maturation [77]. The integration of these somatic cell populations is therefore critical for generating physiologically relevant models of testicular function [70,74].

To achieve these objectives, modern reproductive microphysiological systems increasingly combine advanced biomaterials with microfluidic engineering [78,79]. Compared with conventional two-dimensional cultures, hydrogel scaffolds and extracellular matrix–inspired biomaterials provide three-dimensional structural support that more closely resembles the native tissue environment, enabling improved cell–cell interactions and germ cell differentiation [80,81]. Similarly, three-dimensional organoid and tissue engineering approaches, including emerging bioprinting strategies, enable spatial organization of testicular cell populations and partial reconstruction of seminiferous tubule-like architectures [82]. These approaches are further enhanced by microfluidic organ-on-a-chip platforms capable of delivering nutrients, hormones, and signaling molecules under controlled perfusion while metabolic waste is removed, thereby improving long-term tissue viability and functional maintenance [78].

Control of the biochemical and physical microenvironment represents another critical aspect of *in vitro* spermatogenesis. Within the native testis, oxygen tension, nutrient availability, and hormonal gradients vary across distinct microanatomical regions and contribute significantly to germ-cell development. Reproductive organ-on-a-chip systems increasingly incorporate oxygen-gradient regulation, controlled perfusion systems, and microvascular analogs designed to reproduce these physiological conditions [78,83]. The incorporation of perfusable microvascular networks is particularly important because it enables dynamic nutrient exchange and more accurately reflects the transport processes that occur *in vivo* [83,84].

Beyond structural and physiological reconstruction, emerging platforms are beginning to address the immunological dimensions of testicular function. The testis possesses a unique immune-privileged environment that protects developing germ cells from excessive inflammatory responses while maintaining tissue homeostasis. Reproducing this specialized immune microenvironment remains a significant challenge but may prove essential for the long-term maintenance of functional spermatogenesis in engineered cells [85].

The successful development of *in vitro* spermatogenesis platforms would have profound translational implications. These systems offer opportunities for investigating the mechanisms underlying male infertility, evaluating reproductive toxicants, studying the impact of environmental exposures, and identifying novel therapeutic targets [81]. They may also provide valuable tools for preserving fertility, particularly in prepubertal cancer patients who are unable to produce mature sperm before gonadotoxic treatment [85]. In parallel, human-specific testis-on-a-chip models could facilitate personalized drug screening and improve the prediction of reproductive toxicity during pharmaceutical development [10,78].

Importantly, reproductive microphysiological systems have the potential to reduce reliance on animal experimentation while providing experimental models that more closely reflect human reproductive biology. As these technologies continue to mature, they are expected to become increasingly important platforms for translational reproductive research, precision fertility medicine, and the development of next-generation reproductive therapeutics [11,86].

### 4.3. AI-Integrated Reproductive Chips

While reproductive organ-on-a-chip technologies have significantly advanced the physiological modeling of human reproductive tissues, most current platforms involve largely passive systems. In many experimental settings, environmental parameters such as nutrient composition, oxygen concentration, hormonal supplementation, and waste removal are predefined and manually adjusted according to fixed protocols. However, reproductive tissues are inherently dynamic biological systems that continuously respond to fluctuations in metabolic activity, cellular signaling, and environmental conditions. Consequently, static culture environments may be insufficient to fully replicate the adaptive regulatory mechanisms that govern reproductive physiology *in vivo* [11,87].

Recent advances in biosensing technologies, AI, and microfluidic engineering are creating opportunities to transform organ-on-chip platforms from passive culture devices into intelligent and responsive biological systems. Rather than serving solely as experimental models, next-generation reproductive chips may function as continuously monitored microphysiological environments capable of detecting biological changes in real time and adapting to culture conditions accordingly. This emerging concept represents a shift toward “smart” reproductive bioengineering, in which sensing, computation, and biological regulation become integrated within a unified platform [10,86,87].

Central to this evolution is the incorporation of miniaturized biosensors capable of monitoring multiple physiological parameters simultaneously. Advances in microfabrication now permit real-time assessments of oxygen consumption, pH fluctuations, metabolite production, cellular viability, and, increasingly, hormone-associated secretory dynamics within microfluidic environments. The continuous acquisition of such data provides an unprecedented opportunity to characterize the dynamic behavior of reproductive tissues and developing germ cells throughout culture [87,88].

The growing availability of real-time biological data naturally complements advances in AI and machine learning analytics. AI algorithms are being increasingly applied to identify complex relationships among physiological variables that may be difficult to detect using conventional analytical approaches. By integrating information from biosensors, metabolomic profiling, automated microscopy, and molecular assays, computational systems may generate predictive models capable of anticipating cellular stress, declining tissue function, or alterations in developmental trajectories before overt dysfunction becomes apparent [86,87].

Automated imaging platforms further expand the capabilities of intelligent reproductive chips. High-content imaging systems combined with computer vision algorithms can enable longitudinal monitoring of tissue morphology, cellular organization, germ-cell development, and structural integrity without disrupting the culture environment. Similarly, emerging electrophysiological and bioelectronic sensing technologies may provide additional insights into cellular communication networks and tissue-level responses that are otherwise difficult to measure in real time [89,90,91].

The integration of sensing technologies with AI-driven decision-making introduces the possibility of closed-loop reproductive systems. In such platforms, biological information obtained from the culture environment is continuously analyzed, and the resulting outputs are used to dynamically adjust the experimental conditions. Parameters such as oxygenation, nutrient delivery, hormonal stimulation, perfusion rates, and exposure to cryoprotective agents could be optimized automatically in response to the evolving physiological requirements of the tissue. Such adaptive regulation more closely resembles the homeostatic mechanisms that operate *in vivo* and may improve both experimental reproducibility and biological relevance [86,87,88].

Beyond basic research applications, intelligent reproductive chips could have important translational implications. AI-assisted optimization of culture environments may improve the modeling of infertility, facilitate reproductive toxicology studies, enhance fertility preservation strategies, and support the development of personalized therapeutic interventions. Integration of patient-specific biological data may eventually enable individualized reproductive platforms capable of predicting treatment responses and evaluating therapeutic strategies in silico before clinical implementation [10,87].

Looking further forward, the convergence of organ-on-chip technologies, advanced biosensors, artificial intelligence, and automated laboratory systems may lead to the emergence of autonomous reproductive bioengineering platforms. Although still largely conceptual, such systems would continuously monitor biological processes, integrate multimodal experimental data, and adapt to culture conditions through closed-loop feedback with minimal human intervention. This vision builds on recent advances in microphysiological systems that increasingly incorporate sensing, computation, and system-level integration, as well as emerging frameworks for AI-assisted experimental control and automation in biological research [10,87,92]. While significant technological, regulatory, and ethical challenges remain, these developments illustrate a broader transition from static tissue models toward intelligent, adaptive bioengineering platforms capable of learning, optimization, and predictive biological control. The major reproductive organ-on-chip and microphysiological systems, their current development stages, underlying evidence base, principal applications, and key translational challenges are summarized in Table 3.

While organ-on-a-chip technologies provide physiologically relevant platforms for modeling reproductive tissues and developmental processes, long-term preservation of reproductive potential remains a central challenge in reproductive medicine. Advances in cryobiology have therefore emerged as a complementary field, enabling the preservation of gametes, embryos, and reproductive tissues while supporting fertility preservation, regenerative medicine, and future bioengineering applications [101].

## 5. Cryobiology and Translational Bioengineering

The ability to preserve reproductive cells and tissues across time represents one of the most transformative achievements in modern reproductive medicine. Cryopreservation has become an indispensable component of assisted reproductive technologies (ART), enabling sperm banking, oocyte and embryo storage, fertility preservation, gonadal tissue biobanking, and emerging regenerative medicine applications [12,18]. Beyond its clinical utility, cryobiology has fundamentally altered reproductive healthcare by enabling long-term preservation of reproductive potential across disease treatment, aging, environmental exposure, and changing life circumstances.

Despite substantial advances in cryopreservation technologies, the preservation of reproductive function remains considerably more complex than maintaining cellular survival alone. Reproductive cells and tissues possess highly specialized structural, metabolic, and epigenetic characteristics that render them particularly susceptible to cryoinjury and other environmental perturbations. Consequently, successful cryopreservation must preserve not only membrane integrity and postthaw viability but also mitochondrial function, chromatin architecture, molecular signaling networks, developmental competence, and the capacity to support normal reproductive outcomes. The increasing recognition of these multifaceted requirements has broadened the objectives of cryobiology beyond simple cell survival, promoting a shift toward comprehensive biological preservation that seeks to maintain both functional integrity and molecular integrity across multiple levels of reproductive organization [20,101].

Recent advances in materials science, computational biology, nanotechnology, and bioengineering are accelerating the evolution of cryobiology from preservation technology to multidisciplinary engineering science. Modern cryobiological research increasingly focuses on understanding and controlling the complex cellular and molecular responses that occur during exposure to extreme physicochemical conditions, including thermal stress, osmotic fluctuations, ice formation, and metabolic disruption. Within this framework, successful cryopreservation depends not only on preventing cell death but also on precisely regulating biological state transitions to preserve functional, structural, and molecular integrity. The increasing integration of computational modeling and systems-level analytical approaches is further enabling the development of more predictive preservation strategies capable of optimizing cryopreservation protocols, improving postthaw recovery, and enhancing long-term biological function across diverse cell and tissue types [14,101].

### 5.1. Reimagining Cryopreservation

Traditional cryopreservation strategies are designed primarily to maximize cellular survival following freezing and thawing. Although these approaches have achieved remarkable clinical success, increasing evidence suggests that postthaw viability alone may not accurately reflect the preservation of reproductive function. Cells that appear morphologically intact after thawing may nevertheless exhibit subtle molecular, metabolic, epigenetic, and physiological alterations capable of influencing fertilization potential, embryo development, and long-term biological outcomes [101,102].

The principal challenge arises from the complex cascade of injuries collectively referred to as cryoinjury. During cooling, freezing, storage, and warming, reproductive cells are exposed to multiple sources of stress, including intracellular and extracellular ice crystal formation, osmotic imbalance, membrane phase transitions, oxidative damage, mitochondrial dysfunction, cytoskeletal disruption, and alterations in cellular signaling pathways [16]. These processes are highly interconnected and often continue to evolve after thawing, influencing subsequent cellular behavior and developmental competence.

Particularly important in reproductive medicine is the growing recognition that cryopreservation may influence biological processes that extend beyond immediate cell survival. Oxidative stress generated during cryopreservation can affect sperm chromatin organization, DNA integrity, RNA cargo stability, mitochondrial function, and epigenetic regulation, with potential consequences for fertilization, embryo development, and long-term reproductive outcomes. Moreover, reproductive tissues and gametes frequently exhibit heterogeneous responses to identical preservation protocols owing to differences in cellular composition, metabolic status, developmental stage, age, and underlying physiological conditions. These observations highlight the limitations of traditional one-size-fits-all cryopreservation strategies and underscore the need for more individualized and biologically informed preservation approaches [102,103].

Consequently, contemporary cryobiology is undergoing a transition from empirically optimized protocols toward precision-engineered preservation systems. Rather than focusing solely on temperature control and cryoprotectant exposure, next-generation approaches seek to understand and regulate the complex cellular and molecular responses triggered by cryogenic stress. This emerging paradigm incorporates insights from systems biology, computational modeling, molecular diagnostics, and reproductive physiology to optimize preservation across multiple levels of biological organization and improve both immediate postthaw recovery and long-term functional outcomes [104,105,106].

Within this context, the future of reproductive cryobiology may involve preservation strategies tailored to the unique molecular and functional characteristics of individual cells, tissues, or patients. Such approaches have the potential to improve not only postthaw viability but also reproductive competence, developmental potential, and long-term biological safety. As cryobiology becomes increasingly integrated with artificial intelligence, organ-on-chip technologies, and multiomics analysis, reproductive preservation is evolving from a supportive laboratory procedure to a central component of precision reproductive medicine [103,104].

### 5.2. AI-Guided Cryobiological Optimization

One of the fundamental challenges in cryobiology is the extraordinary complexity of cellular responses to freezing and thawing. The biological consequences of cryopreservation are determined by numerous interdependent variables, including cooling and warming rates, cryoprotectant composition, osmotic dynamics, cellular metabolism, membrane biophysics, oxidative stress responses, and tissue-specific characteristics. Because these variables interact through highly complex and nonlinear mechanisms, the optimization of cryopreservation protocols has historically relied on extensive empirical experimentation, often requiring substantial time, resources, and iterative trial-and-error refinement [101,105,106].

The increasing availability of high-dimensional biological datasets and advances in AI are creating new opportunities to transform cryobiology from an empirically driven discipline into a more predictive and data-guided science. Machine learning algorithms are particularly well suited for identifying complex, nonlinear relationships among biological, physicochemical, and procedural variables that may be difficult to discern using conventional analytical methods. By integrating information from experimental studies, molecular profiling, cellular phenotyping, and preservation outcomes, AI-enabled models can help identify conditions associated with improved cellular survival, functional integrity, and developmental competence while accounting for biological heterogeneity among cells, tissues, and patient populations. These capabilities may accelerate protocol optimization, reduce reliance on trial-and-error experimentation, and support the development of increasingly precise and adaptive preservation strategies [104,106].

In reproductive medicine, AI-assisted cryobiology is emerging as a promising approach for optimizing multiple stages of the preservation process. Computational models can integrate heterogeneous datasets derived from cryoprotectant exposure protocols, thermal profiles, postthaw functional outcomes, molecular biomarkers, and cellular imaging analyses to identify patterns associated with improved preservation performance. Such data-driven frameworks may enable the prediction of optimal cooling rates, warming kinetics, cryoprotectant concentrations, and vitrification conditions tailored to specific cell types, tissues, or clinical contexts. This approach is particularly relevant because spermatozoa, oocytes, embryos, and gonadal tissues exhibit distinct physiological and molecular responses to cryogenic stress, highlighting the need for individualized preservation strategies [104,106].

Beyond protocol optimization, AI may also improve the mechanistic understanding of cryoinjury. Cryopreservation is governed by complex biophysical processes that are often difficult to measure directly, including membrane phase transitions, intracellular ice nucleation, osmotic volume changes, cryoprotectant diffusion dynamics, and oxidative stress propagation. By integrating experimental observations with computational modeling, AI-based systems may help predict critical biological thresholds associated with irreversible cellular damage. These capabilities could facilitate the development of preservation protocols that proactively prevent injury rather than merely minimizing its consequences after it occurs [105,106].

A particularly promising area of development involves the integration of AI with real-time monitoring technologies. Emerging cryobiological platforms increasingly incorporate biosensors, automated imaging systems, and digital quality control tools capable of generating continuous streams of physiological data during freezing and thawing procedures. ML algorithms can analyze these data dynamically, enabling adaptive adjustment of preservation parameters in response to evolving cellular conditions. Such closed-loop approaches introduce the possibility of intelligent cryopreservation systems that continuously optimize preservation conditions throughout the cryogenic process [87,107].

From a translational perspective, AI-guided cryobiology may help address one of the most persistent challenges in reproductive medicine: variability in outcomes among laboratories and clinical centers. Differences in equipment, operator technique, sample characteristics, and protocol implementation often contribute to inconsistencies in postthaw survival and reproductive performance. Predictive computational frameworks capable of standardizing protocol selection, quality assessment, and risk prediction may therefore improve reproducibility across institutions while supporting evidence-based decision-making [104,107].

Ultimately, the convergence of artificial intelligence, computational modeling, and cryobiological engineering is fostering the emergence of predictive cryobiology—a paradigm in which preservation strategies are designed, optimized, and continuously refined through a data-driven understanding of biological responses to cryogenic stress. Such advances may enable future preservation systems that maximize not only postthaw viability but also functional competence, developmental potential, and long-term biological integrity [107,108].

To provide concrete evidence for this discussion, Table 4 summarizes three representative studies in which AI or computational models have been applied to reproductive cryopreservation, spanning sperm extender optimization, human blastocyst warming outcomes, and human sperm bank freezability prediction. These examples illustrate both the promise of the approach and the current limits of the evidence base, including small-to-moderate sample sizes, single-center designs, and, in most cases, an absence of external multisite validation.

### 5.3. Nanowarming and Smart Cryobanking

Although substantial progress has been made in cryopreservation protocols, the warming phase remains among the most vulnerable stages of the preservation process. Cellular injury often occurs not during freezing itself but during thawing, when nonuniform heat transfer can promote ice recrystallization, devitrification, osmotic imbalance, and structural damage. These effects become increasingly significant as cryobiology expands toward the preservation of complex biological systems, including tissues, organoids, and engineered constructs. Within this context, improving the precision and spatial uniformity of warming remains an important area of ongoing research in cryobiological engineering. Emerging nanoparticle-mediated and volumetric heating concepts, developed within the broader field of bioheat transfer, highlight the potential for multiscale thermal control across biological systems and are being explored as strategies to mitigate thermal heterogeneity during thawing [112,113].

Among the most promising advances is the development of nanowarming technologies. Unlike conventional warming approaches that rely primarily on external heat transfer, nanowarming utilizes biocompatible magnetic nanoparticles distributed throughout the preserved sample. Upon exposure to alternating magnetic fields, these nanoparticles generate heat internally, enabling rapid and more spatially uniform warming of the biological material. This approach can reduce thermal gradients and devitrification-associated injury and has been associated with improved preservation outcomes in experimental reproductive models [13,14].

In addition to nanowarming, complementary experimental approaches such as laser-based and photothermal warming are being investigated to improve the spatial control of energy delivery during thawing and reduce thermal heterogeneity. These strategies reflect ongoing efforts to address fundamental limitations in heat transfer during the rewarming of cryopreserved biological systems, particularly as applications extend toward tissues and complex multicellular constructs [113,114]. In parallel, cryobanking is undergoing a digital transformation from passive storage to sensor-enabled infrastructure. Modern biobanking systems increasingly incorporate advanced monitoring and cryopreservation technologies to improve the long-term preservation, management, and reliability of stored reproductive materials [12]. AI further supports these developments through predictive maintenance and anomaly detection, enabling improved quality assurance and risk mitigation in long-term biological storage systems [12,26,115]. Collectively, these advances suggest a gradual convergence of enhanced thermal control strategies and digitally integrated cryobanking systems, shifting preservation frameworks toward more data-driven and reliability-focused infrastructures.

## 6. Intelligent Reproductive Preservation and Developmental Integrity in Precision Reproductive Medicine

Reproductive medicine is increasingly shifting from conventional storage and retrieval systems toward intelligent preservation systems in which gametes, embryos, tissues, and engineered constructs are integrated with digital monitoring and decision-support platforms. This emerging framework positions biobanking as a dynamic component of precision reproductive healthcare, linking cryobiology, artificial intelligence, and bioengineering [101,115].

In parallel, assisted reproductive technologies have expanded clinical success in infertility treatment, prompting a broader definition of outcomes that extends beyond fertilization and live birth to include the preservation of developmental integrity. This concept provides evidence that early reproductive stages constitute critical windows of epigenetic programming, during which molecular networks governing differentiation, metabolism, and long-term function are established. Because these processes are highly sensitive to environmental and procedural perturbations, reproductive interventions may influence developmental trajectories in ways that are not captured by standard clinical endpoints [2,3].

### 6.1. From Clinical Success to Developmental and Epigenetic Integrity

The long-term trajectory of reproductive medicine is shifting beyond traditional outcomes such as fertilization, implantation, and live birth toward a broader definition of success that includes developmental and molecular integrity. This emerging framework emphasizes the preservation of the epigenetic and cellular programs that regulate normal development across the lifespan [116,117]. This shift is supported by developmental biology, which identifies gametogenesis, fertilization, and preimplantation development as highly sensitive windows of epigenetic programming. During these stages, extensive epigenetic remodeling establishes regulatory networks governing differentiation, metabolism, and tissue development. Because these processes are highly dynamic and environmentally responsive, assisted reproductive procedures could influence epigenetic and developmental trajectories; however, human evidence remains heterogeneous, and observed epigenetic differences cannot always be clearly distinguished from the effects of parental subfertility or other confounding factors [118].

A key conceptual foundation is the developmental origins of the health and disease (DOHaD) paradigm, which links periconception environmental conditions to lifelong health outcomes. Mechanistically, this involves coordinated regulation through DNA methylation, histone modifications, chromatin organization, higher-order genome architecture, and noncoding RNAs, which collectively shape developmental gene expression programs [1,2]. However, evidence linking ART specifically to persistent epigenetic alterations in humans remains limited and heterogeneous, with some reported differences being small and potentially influenced by parental subfertility and other clinical or environmental factors [118].

### 6.2. Cryopreservation, Cellular Stress, and Epigenomic Vulnerability

Cryopreservation is among the most widely used yet biologically complex interventions in assisted reproductive technologies. Although vitrification and optimized protocols have improved post-thaw survival, accumulating evidence shows that morphological viability alone does not ensure the preservation of molecular fidelity or developmental competence [2,101]. During cooling, storage, and warming, reproductive cells undergo interconnected stresses—including osmotic imbalance, membrane phase transitions, mitochondrial dysfunction, and reactive oxygen species (ROS) generation—that can propagate across cellular networks and disrupt chromatin organization, gene regulation, and metabolic homeostasis [101]. Oxidative stress, in particular, has been implicated in epigenomic instability through effects on DNA methylation, histone modification, RNA stability, and mitochondrial signaling. Spermatozoa are especially vulnerable because of their limited cytoplasmic repair capacity and compact chromatin architecture, although similar susceptibility is observed in oocytes, embryos, and gonadal tissues [119,120]. Collectively, these findings indicate that cryopreservation stress may extend beyond acute cellular injury to include molecular, epigenetic, and functional perturbations, motivating a shift toward integrity-centered cryobiology in which success is defined by the preservation of molecular stability, developmental potential, and reproductive competence [2,101].

### 6.3. Toward Epigenetic-by-Design Reproductive Systems

Assisted reproductive technologies have historically been evaluated using clinical endpoints such as the fertilization rate, embryo morphology, implantation success, and live birth rate. However, evidence from developmental biology and epigenetics indicates that these measures may not fully capture subtle molecular and epigenetic perturbations occurring during early developmental windows [118,121]. This has led to growing interest in preserving developmental fidelity throughout the reproductive workflow. The concept of epigenetic-by-design reproductive systems extends this perspective by embedding epigenetic and developmental considerations directly into the design of reproductive technologies rather than treating them as retrospective quality metrics [118,122]. Operationally, an epigenetic-by-design framework would require predefined molecular indicators of developmental integrity rather than treating epigenetic preservation as a purely conceptual objective. Candidate measures could include locus- or genome-wide DNA methylation profiling, assessment of genomic imprinting regions, selected histone or chromatin-associated markers, and transcriptomic indicators of developmental competence. Such measurements could be incorporated as research-stage quality-control endpoints to compare culture, cryopreservation, or manipulation conditions and to identify procedures associated with reproducible molecular deviations. At present, however, no universally validated biomarker, threshold, or clinical decision rule has been established for defining an “epigenetically optimal” gamete or embryo, and these measures should therefore be considered investigational rather than routine clinical criteria [118].

The implementation of this framework will depend on continued advances in microfluidic technologies, physiomimetic culture systems, and next-generation embryo production platforms that more closely reproduce the native reproductive microenvironment while reducing handling-related stress during gamete and embryo manipulation [123,124]. In parallel, integrated sensing and monitoring technologies are enabling increasingly precise assessments of reproductive-cell function, including oxygen consumption, metabolic activity, biomechanical properties, and other indicators of cellular homeostasis, that may provide noninvasive measures of developmental competence and embryo quality [124,125].

AI is expected to play a central role when multimodal datasets derived from imaging, molecular profiling, and laboratory monitoring data are integrated. These systems may enable adaptive optimization of culture and preservation conditions, creating closed-loop reproductive environments capable of responding dynamically to biological feedback signals [5]. In a future epigenetic-by-design framework, such systems could incorporate validated molecular integrity measures as additional monitoring variables; however, the establishment of clinically meaningful thresholds and intervention rules remains important.

### 6.4. Integrated Perspective: Toward Precision Reproductive Preservation

Advances across cryobiology, developmental biology, epigenetics, and bioengineering are collectively driving a transition toward precision reproductive preservation. In this emerging paradigm, reproductive technologies are evaluated not only by efficiency or survival outcomes but also by their ability to preserve molecular fidelity and developmental resilience [126]. This shift reflects a broader move toward integrity-centered reproductive medicine, in which the objective is to maintain biological systems in a state compatible with normal developmental programming and long-term health. Within this framework, reproductive preservation becomes a continuous, data-informed process that integrates molecular, functional, and systems-level information to guide both technological design and clinical application [127].

## 7. Clinical Translation and Emerging Applications

The convergence of AI, reproductive bioengineering, organ-on-chip technologies, precision cryobiology, and developmental monitoring is creating opportunities to redefine how reproductive health is diagnosed, managed, and preserved. Recent advances, such as AI-enabled reproductive organoid and organ-on-chip platforms, placenta-on-chip and fetal–maternal interface models [11,128], and high-throughput reproductive tissue-on-chip systems [23], illustrate early translational interest in these technologies, although clinical implementation has not yet been achieved. Although many remain in early stages of development, their integration may transform reproductive medicine from a predominantly reactive discipline into a predictive, personalized, and adaptive healthcare framework, with implications extending beyond infertility treatment to reproductive toxicology, regenerative medicine, biotechnology, and population health.

Notably, to date, none of the integrated technologies discussed in this section—including AI-driven organ-on-chip systems, precision cryobiology platforms, and digital reproductive twins—have been evaluated in registered clinical trials, prospective implementation studies, or multicenter validation cohorts, nor have they received regulatory approval for clinical use. The discussion below therefore describes translational trajectories and proof-of-concept advances rather than clinical implementation or patient outcome benefits.

One of the most immediate applications is precision fertility diagnostics. By integrating advanced imaging, molecular profiling, functional biomarkers, and clinical data, AI-based systems may detect subtle reproductive dysfunction before clinical manifestations emerge, enabling earlier intervention, improved patient stratification, and more individualized treatment selection. Similarly, AI-assisted sperm analysis, automated sperm selection, and intelligent embryo assessment platforms may reduce operator-dependent variability and improve reproducibility across assisted reproductive technology laboratories [45].

The combination of organ-on-a-chip systems with patient-derived biological data introduces the possibility of personalized reproductive models capable of simulating reproductive physiology, evaluating therapeutic responses, assessing environmental exposures, and optimizing fertility preservation strategies. Integration with digital twin technologies may further support individualized risk assessment and treatment planning [129].

Advances in cryobiology are likewise driving the development of intelligent preservation platforms incorporating biosensors, predictive analytics, and automated quality-control systems. Such approaches may enhance the safety, scalability, and long-term reliability of reproductive biobanking for gametes, embryos, reproductive tissues, organoids, and engineered reproductive constructs [12].

Beyond clinical applications, human-relevant organ-on-chip platforms and AI-enabled analytical tools may improve the study of reproductive disorders, endocrine disruption, environmental toxicology, and developmental programming while reducing the reliance on animal models and increasing the translational relevance of preclinical research [9]. These advances are particularly important for preserving fertility in oncology patients, where improved cryobiological, tissue engineering, and computational approaches may enhance long-term reproductive outcomes following gonadotoxic therapies [130].

Collectively, these developments reflect a transition from isolated reproductive procedures toward integrated biological systems capable of prediction, adaptation, and continuous learning. As AI, bioengineering, and systems biology become increasingly interconnected, the boundaries between diagnosis, treatment, preservation, and long-term reproductive health monitoring may progressively dissolve, establishing a new paradigm for precision reproductive medicine.

### Industrial and Pharmaceutical Transformation of Reproductive Medicine

The convergence of artificial intelligence, reproductive bioengineering, organ-on-chip technologies, and advanced cryobiology extends reproductive medicine beyond fertility care to broader applications in biotechnology, drug discovery, toxicology, and regenerative medicine. Once primarily focused on assisted reproduction and fertility preservation, the field is increasingly positioned as a translational platform within the emerging bioeconomy [65].

A key driver of this transformation is the increasing availability of high-dimensional reproductive datasets generated from imaging, molecular profiling, laboratory monitoring, and clinical records. When integrated with artificial intelligence, these data enable biomarker discovery, predictive modeling, and the development of digital reproductive-health platforms, facilitating the translation of fertility technologies into data-driven clinical applications and translational decision-support systems [4,5].

The pharmaceutical implications are particularly significant. Reproductive drug development has historically been constrained by the limited predictive value of animal models and conventional cell cultures, which fail to fully recapitulate human reproductive physiology. Human-relevant organ-on-a-chip and microphysiological systems offer a promising alternative by enabling more accurate modeling of endocrine regulation, gametogenesis, implantation, reproductive toxicology, and disease mechanisms. These platforms may increase translational efficiency, improve screening of endocrine-disrupting compounds, and support the identification of therapeutic targets for infertility and reproductive disorders [131].

AI further enhances these capabilities by integrating organ-on-chip, multiomics, and clinical datasets to enable biomarker discovery, the prediction of therapeutic responses, and the optimization of precision reproductive interventions [132]. These developments position reproductive medicine as an increasingly important component of the emerging bioeconomy. As reproductive technologies converge with pharmaceutical sciences, biomedical engineering, and digital health, they are evolving from specialized clinical tools to enabling platforms that drive innovation across the life sciences.

Despite this translational potential, several concrete barriers currently limit the industrial and pharmaceutical adoption of AI-integrated reproductive organ-on-chip and cryobiological platforms. Reproducibility and cross-laboratory standardization remain unresolved: interlaboratory validation of organ-on-chip assays is hindered by variability in cell sourcing, culture protocols, and analytical endpoints, and no reproductive-specific microphysiological system has yet achieved the endpoint harmonization required for routine industrial use [133]. Throughput and scalability are similarly constrained, as most reproductive organ-on-chip and AI-integrated platforms remain manually operated, low-throughput research prototypes rather than industrially scalable systems suitable for high-volume pharmaceutical screening [134,135]. Regulatory qualification is at an early stage: although the FDA Modernization Act 2.0 and subsequent FDA/NIH roadmaps have opened a formal pathway for organ-on-chip systems and other new approach methodologies (NAMs) to be qualified for preclinical and drug-safety evaluation, no organ-on-chip platform—reproductive or otherwise—has yet achieved full qualification through the FDA’s Innovative Science and Technology Approaches for New Drugs (ISTAND) program, indicating that reference compound panels, validation packages, and acceptance criteria specific to reproductive applications remain to be established [136]. Intellectual property and data-access constraints add a further translational barrier: reproductive datasets used to train AI models are frequently proprietary to individual fertility clinics or commercial vendors, and reliance on proprietary, noninterpretable algorithms limits independent validation, cross-platform benchmarking, and transparent regulatory review [137]. Finally, cost remains a substantial obstacle to scalability and equitable access: assisted reproductive procedures already carry a high per-cycle cost, and the incremental expense of GMP-grade organ-on-chip manufacturing, biosensor integration, and AI infrastructure has not yet been demonstrated to be economically viable at the industrial scale. Addressing these gaps—through standardized validation frameworks, multisite reproducibility testing, transparent and auditable algorithms, and formal regulatory qualification pathways specific to reproductive applications—will be necessary before the industrial and pharmaceutical potential described above can be realized in practice.

## 8. Regulatory and Ethical Challenges in AI-Driven Reproductive Biotechnology

The rapid expansion of artificial intelligence–enabled reproductive technologies is transforming reproductive medicine into a highly data-intensive and algorithmically mediated field, raising complex regulatory and ethical challenges alongside clinical opportunities [4]. A central concern is the increasing reliance on AI for embryo assessment, gamete evaluation, and laboratory decision support. While these systems may enhance predictive accuracy and operational efficiency, limitations related to transparency, interpretability, dataset bias, and fairness remain significant. Without robust validation and oversight, such issues may contribute to inequities in reproductive outcomes across populations.

To address these methodological and reporting limitations, clinical AI evaluation must be grounded in established international reporting frameworks. Standardized reporting protocols—specifically TRIPOD+AI for multivariable prediction models, DECIDE-AI for early-stage clinical implementation, and CONSORT-AI for clinical trials—are critical to ensure algorithmic transparency, reproducibility, and robust validation of clinical readiness in precision reproductive medicine.

The digitization and continuous monitoring of reproductive processes further introduce critical questions regarding ownership, consent, privacy, and secondary use of highly sensitive embryonic, genetic, and developmental data. As reproductive datasets become increasingly embedded within commercial and research-oriented AI ecosystems, comprehensive data governance frameworks are needed to ensure accountability, transparency, and public trust [4].

In parallel, regulatory oversight is evolving rapidly to address algorithmically driven medical devices. Jurisdictional frameworks—such as the European Union Artificial Intelligence Act (EU AI Act), the EU *In Vitro* Diagnostic Regulation (IVDR, Regulation EU 2017/746), and the U.S. FDA Guidance on Software as a Medical Device (SaMD) and AI/ML-based software functions—may subject certain clinical AI systems to high-risk or other regulated medical device requirements, depending on their intended use, functionality, and jurisdiction. Compliance with these frameworks requires rigorous postmarket surveillance, continuous algorithmic auditability, and clear boundaries between supportive algorithms and clinical oversight. Emerging capabilities in embryo selection, developmental monitoring, and potential germline-related interventions further intensify ethical concerns surrounding the boundary between therapeutic intervention and biological enhancement. These issues are compounded by uncertainty regarding the long-term developmental and epigenetic consequences of reproductive technologies, which may extend across multiple life stages and potentially across generations.

Addressing these challenges will require strengthened international regulatory coordination as reproductive technologies evolve into globally distributed biotechnological platforms. Harmonized standards involving regulatory authorities, professional societies, and international organizations are essential to ensure safety, efficacy, data protection, and ethical compliance. Future governance frameworks should integrate algorithmic accountability, biological safety assessment, reproductive data governance, and the continuous validation of AI systems in reproductive settings. Such a multilayered approach would support a transition from reactive regulation toward anticipatory, systems-level oversight capable of adapting to rapidly evolving technologies. Collectively, these measures will be essential for preserving ethical integrity; safeguarding developmental health; and ensuring that the convergence of AI and reproductive biotechnology remains aligned with clinical, societal, and bioethical priorities.

## 9. Future Directions and Technology Roadmap for Precision Reproductive Bioengineering (2026–2045)

Building on the technological, clinical, and regulatory developments discussed throughout this review, reproductive biotechnology may evolve toward increasingly integrated and data-driven systems over the next two decades, provided that current technical, regulatory, and clinical validation challenges are successfully addressed. Rather than advancing as isolated innovations, artificial intelligence, organ-on-chip technologies, precision cryobiology, multiomics profiling, and digital health platforms could converge into interconnected reproductive ecosystems supporting diagnosis, preservation, therapeutic development, and long-term health monitoring—although this convergence is contingent on validation outcomes, regulatory acceptance, demonstrated clinical benefit, interoperability between platforms, and cost-effective scalability at each stage. To provide a structured overview of this anticipated—but not guaranteed—evolution, the major technological milestones, tentative timelines, and projected stages of implementation are summarized in Table 5. The timelines shown represent one plausible trajectory under favorable conditions rather than a deterministic forecast; progression from one stage to the next for any given technology is not assumed and may be delayed, altered, or halted by technological failure, regulatory rejection, insufficient demonstrated clinical benefit, interoperability barriers, or unresolved cost constraints.

Near-Term (2026–2030): Digitalization and Clinical Standardization of Reproductive Assessment

The near-term phase will likely be characterized by broader adoption of AI-assisted reproductive diagnostics, automated laboratory workflows, and digitally enabled cryobanking systems. Advances in data integration and multimodal fertility assessment may improve diagnostic precision, standardization, and clinical decision support while establishing the digital infrastructure required for more advanced predictive technologies [4].

Mid-Term (2030–2035): Predictive Modeling and Organ-on-Chip Integration

During the mid-term period, continued progress in organ-on-chip platforms, multiomics technologies, and computational modeling may enable increasingly personalized approaches to reproductive medicine. Digital reproductive twins and patient-specific computational models may eventually support the simulation of fertility trajectories, therapeutic responses, and preservation strategies, although such applications remain largely investigational and require substantial validation before clinical implementation [131].

Long-Term (2035–2045): Autonomous and Space-Enabled Reproductive Bioengineering Ecosystems

If current technological trajectories continue, the long-term horizon may involve increasingly automated reproductive platforms integrating robotics, biosensors, artificial intelligence, and continuous quality control systems, with autonomous functions potentially supporting selected laboratory tasks under appropriate human oversight. Such systems could enable adaptive optimization of reproductive workflows and more comprehensive monitoring of developmental and epigenetic outcomes. In parallel, reproductive bioengineering may expand into extreme environments, including potential applications relevant to assisted reproductive technologies and reproductive health in long-duration space exploration, where microgravity and radiation pose significant challenges to gametogenesis, endocrine regulation, and embryonic development [139,140].

Collectively, this roadmap suggests a gradual transition from AI-augmented clinical tools toward integrated reproductive bioengineering ecosystems in which biological, computational, and engineering components operate within unified frameworks.

## 10. Translational Requirements and Future Readiness of Precision Reproductive Medicine

The realization of the technological roadmap outlined above depends on requirements that extend beyond technological development itself. Translation into precision reproductive medicine will require rigorous biological and clinical validation, standardized performance metrics, interoperable data infrastructures, developmental safety assessment, ethical governance, and appropriate regulatory frameworks [2,54,65,127]. This section therefore focuses on the conditions required to move emerging technologies from experimental and predictive platforms toward clinically reliable and responsibly governed applications.

### 10.1. Integration of Emerging Reproductive Technologies

The clinical translation of AI, reproductive bioengineering, organ-on-chip systems, precision cryobiology, multiomics, and developmental monitoring will require interoperable infrastructures capable of integrating biological, computational, and clinical data across the reproductive continuum [65,127,133]. Such integration should allow information generated from diagnostic platforms, experimental models, cryopreservation systems, and longitudinal monitoring to inform clinical decision-making while maintaining data quality, traceability, and compatibility across platforms. Standardized data structures and performance metrics will therefore be essential for reproducible implementation across institutions.

### 10.2. Developmental Integrity and Translational Validation

A central requirement for next-generation reproductive medicine is ensuring that technological innovation remains aligned with long-term developmental safety. Because reproductive interventions occur during highly sensitive windows of biological and epigenetic programming, evaluation frameworks must extend beyond procedural success and short-term clinical outcomes to include developmental integrity, biological fidelity, and long-term health trajectories. Achieving this objective will require rigorous preclinical and clinical validation, standardized performance metrics, reproducibility assessment, external and multicenter validation, and longitudinal monitoring [2,118,127]. These requirements are particularly important for AI-based systems and engineered reproductive models, for which predictive performance alone does not establish clinical validity or developmental safety.

### 10.3. Building the Infrastructure for Precision Reproductive Medicine

The principal challenge facing the field is not only the development of individual technologies but also their translation into coordinated healthcare systems. Progress will depend on interdisciplinary collaboration among reproductive biologists, clinicians, bioengineers, computational scientists, and regulatory stakeholders, alongside multicenter validation networks, interoperable data ecosystems, longitudinal developmental registries, and appropriate regulatory and ethical frameworks [54,65,133]. Successful translation will therefore require infrastructures capable of supporting continuous evaluation, postimplementation monitoring, transparency, accountability, and equitable access. Ultimately, the readiness of precision reproductive medicine depends on whether emerging technologies can be incorporated into clinically reliable, ethically governed, and continuously learned healthcare systems while safeguarding developmental health across generations.

### 10.4. Methodological Limitations and Implementation Priorities

While the converging technologies outlined in Section 9 present a transformative horizon for ART, realization of this roadmap faces critical evidence-based, scope, and operational constraints. Addressing these limitations defines the mandatory scientific prerequisites for safe clinical translation. Currently, most AI-assisted diagnostic and predictive models rely heavily on single-center, retrospective datasets, which restricts their generalizability across various clinical protocols, diverse patient demographics, and heterogeneous imaging platforms [19,29,58]. Future research should therefore prioritize prospective, multicenter, and externally validated studies using independent testing cohorts, standardized reporting frameworks, and prespecified subgroup analyses to assess model robustness across diverse patient populations and clinical settings. Prospective clinical trials and long-term follow-up studies will also be essential to establish clinical effectiveness, safety, and sustained outcomes before widespread implementation. This issue is further compounded by substantial outcome heterogeneity across the literature—such as nonstandardized definitions for embryo viability, live-birth endpoints, and sperm functional parameters—which continues to impede robust cross-study synthesis and quantitative meta-analysis. Parallel translational gaps exist in microfluidic bioengineering; microphysiological platforms (organ-on-chip) and advanced cryopreservation protocols, such as nanowarming, remain largely restricted to preclinical validation [20,112,123]. Consequently, robust longitudinal human data evaluating the long-term developmental, metabolic, and epigenomic safety of these interventions remain sparse [2,118,127]. Furthermore, the scope of this review inherently introduces methodological boundaries. As an integrative narrative synthesis prioritizing broad systems-level mapping over the granular quantitative pooling of a systematic review, the projected 2026–2045 technology roadmap (Table 4) should be interpreted as directional projections rather than deterministic forecasts.

To bridge these empirical and methodological limitations and enable the long-term milestones of precision reproductive bioengineering, immediate research and governance frameworks must focus on six core implementation priorities. First, the field requires standardized benchmarking frameworks, including open-access, multicenter validation datasets with harmonized outcome definitions, to rigorously evaluate algorithmic performance and microfluidic utility across diverse clinical environments [65]. Second, establishing longitudinal epigenetic registries is critical for tracking prospective cohorts of offspring conceived via AI-selected gametes or microfluidic culture systems, thereby monitoring long-term health and transgenerational stability [118]. Third, predeployment explainability and bias auditing must become regulatory prerequisites, ensuring algorithmic transparency through interpretable decision features and verifying performance parity across demographic and clinical subgroups prior to commercial integration [54]. Fourth, regulatory science must adapt by establishing dynamic oversight pathways capable of continuous postmarket surveillance and iterative reapproval mechanisms for continuously learning, adaptive AI models. Fifth, considerations of global health equity must guide future development; explicitly evaluating implementation feasibility, scalability, and cost-effectiveness in low- and middle-resource settings is essential to prevent advanced bioengineering tools from exacerbating existing global disparities in reproductive healthcare. Finally, targeted, domain-specific systematic reviews and meta-analyses addressing isolated subtechnologies—such as standalone AI embryo scoring or individual organ-on-chip models—are needed to complement this integrative overview with focused, quantitative statistical rigor.

## 11. Conclusions

Reproductive medicine is entering a phase of profound technological convergence in which artificial intelligence, reproductive bioengineering, organ-on-chip systems, precision cryobiology, and developmental epigenetics are being increasingly integrated. Emerging quantitative evidence indicates that the transition toward predictive, personalized, and data-driven reproductive medicine has begun, although the strength and clinical relevance of evidence vary across technologies and applications. These advances have the potential to improve fertility assessment, optimize assisted reproductive procedures, enhance cryopreservation strategies, and deepen the mechanistic understanding of gamete biology and embryonic development. They may also provide the foundation for translational infrastructures that integrate diagnosis, prediction, ex vivo experimentation, preservation, and long-term developmental monitoring within increasingly connected clinical ecosystems.

Nevertheless, technological innovation alone cannot ensure meaningful clinical transformation, and strong computational or experimental performance does not necessarily translate into improved patient outcomes. Persistent challenges related to biological complexity, epigenetic and developmental safety, reproducibility, multicenter validation, data interoperability, regulatory governance, cost-effectiveness, and long-term clinical outcomes continue to limit translation into practice. Addressing these barriers will require rigorous prospective validation, standardized evaluation, ethical oversight, and longitudinal monitoring to establish whether emerging technologies provide reproducible and clinically meaningful benefits.

Looking forward, the future of reproductive medicine may be defined by increasingly interconnected ecosystems in which biological and computational intelligence operate synergistically. The pace and extent of this transformation depend on demonstrated safety, reproducibility, clinical benefit, scalability, and equitable applicability rather than technological capability alone. Technologies that satisfy these requirements may contribute to a new generation of precision reproductive care, while those that fail to demonstrate sufficient validity or clinical value may remain investigational or undergo limited adoption. Ultimately, the responsible integration of technological innovation with rigorous evidence and clinical oversight will be essential to advancing reproductive healthcare while safeguarding developmental integrity across future generations.

## Figures and Tables

**Table 1:** Comparison of the scope and thematic coverage of the present review with those of representative recent reviews in precision reproductive medicine.

Representative Recent Review	AI/ML	OOC/MPS	Cryobiology/Fertility Preservation	Multiomics	Digital Twins	Developmental/Epigenetic Safety	Integrated Translational Framework
Wu et al. (2025) [21]—Artificial intelligence and assisted reproductive technology: A comprehensive systematic review	✓	—	—	*	—	*	—
Pan et al. (2025) [22]—Organ-on-a-Chip Models of the Female Reproductive System: Current Progress and Future Perspectives	*	✓	*	*	—	*	*
Truong et al. (2025) [23]—Modeling reproductive and pregnancy-associated tissues using organ-on-chip platforms	*	✓	*	*	—	*	*
Voros et al. (2025) [24]—Epigenetic Alterations in Ovarian Function and Their Impact on Assisted Reproductive Technologies: A Systematic Review	—	—	*	✓	—	✓	—
Vallée et al. (2026) [25]—Digital twins in fertility, assisted reproductive technology and pregnancy: A systematic review	✓	*	*	*	✓	*	*
Lei et al. (2026) [26]—The application of artificial intelligence in cryopreservation: Technological advances and future challenges	✓	—	✓	*	—	*	*
Zhang et al. (2026) [8]—Ovarian tissue quality assessment and fertility preservation strategies enabled by multiomics and artificial intelligence	✓	—	✓	✓	—	*	*
Present review	**✓**	**✓**	**✓**	**✓**	**✓**	**✓**	**✓**

**Note:** ✓ indicates a major or explicit focus of the review; * indicates secondary or partial emphasis; and — indicates that the topic is not a substantive focus.

**Table 2:** Current Applications of AI in Reproductive Medicine.

Technology	Current Readiness Level	Advantages	Limitations
AI-assisted semen analysis	Clinical implementation	Rapid, objective sperm assessment	Limited standardization
AI-assisted sperm functional phenotyping	Translational	Integrates SDF, oxidative stress, mitochondrial function	Requires large datasets
AI-based fertility outcome prediction	Translational/clinical	Personalized treatment planning	Dataset bias, class imbalance, limited external validation, and model generalizability
Multiomics fertility diagnostics	Experimental/translational	Comprehensive reproductive profiling	Data integration complexity
AI-integrated reproductive chips	Experimental	Real-time adaptive monitoring	Technical complexity
Reproductive digital twins	Emerging/conceptual	Personalized reproductive simulation	Requires longitudinal data
Autonomous reproductive laboratories	Conceptual	Closed-loop optimization	Regulatory and ethical barriers

**Table 3:** Organ-on-Chip and Microphysiological Systems in Reproductive Bioengineering: Development Stage and Evidence Base.

Model	Development Stage	Evidence Base/Species	Primary Applications	Current Challenges	Representative References
Testis-on-chip	Advanced experimental (preclinical)	Predominantly animal models, including mouse and porcine systems; limited human validation	Spermatogenesis modeling, toxicology screening, fertility preservation	Long-term maintenance of spermatogenesis and reproducibility of tissue function	(Kanbar et al., 2022 [81]; Komeya et al., 2016, 2017 [69,93])
Ovary-on-chip	Experimental	Primarily experimental models	Folliculogenesis, endocrine studies, drug screening	Hormonal cycle reproduction	(He, 2017 [94]; Xiao et al., 2017 [95])
Endometrium-on-chip	Experimental	Experimental human/organotypic models	Implantation biology and embryo-endometrium interactions	Dynamic hormonal responsiveness	(Gnecco et al., 2017 [96]; Xiao et al., 2017 [95])
Placenta-on-chip	Advanced experimental	Experimental human/animal models	Maternal-fetal transport and developmental toxicology	Reproducing complex vascular interfaces	(Blundell et al., 2016 [97]; Lee et al., 2016 [98])
Reproductive tract-on-chip	Emerging	Experimental and conceptual models	Gamete transport and fertilization studies	Multiorgan integration	(Bhatia & Ingber, 2014 [99]; Xiao et al., 2017 [95])
Multiorgan reproductive systems	Conceptual/translational	Primarily conceptual and early experimental systems	Reproductive axis simulation	Standardization and scalability	(Edington et al., 2018 [100]; Ingber, 2022 [10])

**Table 4:** Representative Studies Applying AI/Computational Models to Reproductive Cryopreservation.

Parameter	Tu et al. (2022) [109]—Bull Sperm Extender Optimization	Conversa et al. (2025) [110]—Vitrified Human Blastocysts	Jiang et al. (2017) [111]—Human Sperm Bank Freezability
Reproductive material evaluated	Bull (bovine) spermatozoa	Human vitrified–warmed blastocysts	Human semen (sperm bank samples)
Sample size and data source	68 Holstein bulls; 200 extender–postthaw motility pairs (training) and 32 pairs (test) across 21 experimental generations; independent field validation in 15 additional bulls	846 vitrified blastocysts (815 transferred), single-center retrospective cohort, Feb 2022–May 2024 (IVIRMA/IVI Valencia)	180 semen samples from a single human sperm bank
Input variables	Concentrations of 11–12 extender components (cryoprotectants, sugars, antioxidants, buffer, egg yolk, membrane stabilizers)	Pre/postvitrification embryologist (ASEBIR) morphology grade, AI-derived image quality score, postwarming blastocoel contraction pattern/timing, patient age, BMI, oocyte source, day of blastulation, PGT-A status, cycle type	Basal semen parameters, including CASA-derived kinetic measures (progressive motility, straight-line velocity, average path velocity)
Algorithm/model used	Artificial neural network and Gaussian process regression, coupled with a differential evolution search algorithm	Commercial image-based AI model (Embryo Predict, Alife Health) plus a separately trained artificial neural network classifier	Multiple logistic regression with backward elimination variable selection
Predicted outcome	Postthaw sperm motility (total and progressive)	Ongoing pregnancy/implantation outcome (fetal heartbeat at week 12)	Postthaw sperm “freezability” (good vs. poor quality after cryopreservation)
Validation strategy	10-fold cross-validation; held-out generation-6 test set; prospective field validation of top algorithm-derived extenders against control in an independent bull cohort	Multivariable logistic regression with clinical covariates; 70/30 train–test split for the neural network classifier	ROC curve analysis; prospective second-stage validation in which model-selected samples were frozen–thawed and outcomes compared to first-stage data
Performance metrics	Cross-validated MSE 525 (ANN) vs. 576 (GPR); postthaw motility improved from 25.5% (generation 1) to up to 68.3% (generation 21)	Neural network classifier AUC 0.656; AI score odds ratio 1.29 (95% CI 1.13–1.47, *p* < 0.001) in multivariable model	AUC 0.789 for the combined model vs. 0.746–0.612 for individual parameters alone; good-freezability rate rose from 67% to 94% (*p* = 0.003) with model-guided selection
Comparison with conventional methods	Algorithm-optimized media were statistically comparable to a commercial extender refined over ~60 years of empirical optimization, achieved with far fewer trials than a full factorial design	AI and conventional embryologist morphology scoring were both significantly associated with outcome; the AI score added independent predictive value alongside standard morphological grading	Outperformed conventional single-parameter WHO semen-quality thresholds used in isolation
Principal limitations	Gains seen during optimization were not always replicated in follow-up field trials; only comparable to, not clearly superior to, industry-standard extender; requires a true field-fertility trial for full validation	AI algorithm trained externally on fresh (not postthaw) embryo images at a fixed time point; developers had no access to this clinic’s data or labels; single-center retrospective design; modest AUC indicates limited discriminative power	Single-center, single-population dataset; logistic regression rather than a genuine machine learning architecture, so nonlinear interactions may be undercaptured; no external multisite validation reported

**Table 5:** Technology Roadmap for Reproductive Bioengineering (2026–2045). Projected evolution is based on current advances in artificial intelligence, reproductive bioengineering, cryobiology, and microphysiological systems (Abtahi et al., 2023 [104]; Ingber, 2022 [10]; Leung et al., 2022 [107]; Vunjak-Novakovic et al., 2021 [138]; Zhao et al., 2024 [87]). The timelines and milestones below are conditional projections rather than confirmed outcomes; each stage assumes successful completion of the validation, regulatory, and interoperability requirements outlined for the preceding stage.

Technology	Current Level of Evidence	Conditional Projected Trajectory	Required Validation and Measurable Success Criteria	Technical and Regulatory Dependencies	Level of Uncertainty
AI-assisted semen analysis	Emerging evidence supports AI-based sperm detection, classification, and quantitative assessment, but performance and generalizability vary across datasets, devices, and laboratories.	2026–2030: multicenter validation and possible selective clinical adoption. 2030–2035: broader standardization if reproducibility is demonstrated. 2035–2045: potentially more autonomous monitoring if sustained reliability is established.	Prospective multicenter validation; reproducibility across laboratories and platforms; predefined diagnostic-performance thresholds; evidence that AI improves assessment consistency or clinically relevant decision-making.	Representative training datasets; standardized imaging and laboratory protocols; interoperability; cybersecurity; regulatory clearance and appropriate laboratory oversight.	Moderate–high
AI-assisted embryo assessment	Retrospective and early prospective studies suggest potential for automated embryo assessment, but evidence linking AI scores to clinically meaningful outcomes remains limited and heterogeneous.	2026–2030: possible use as decision-support in selected IVF settings. 2030–2035: adaptive scoring may develop if outcome-linked validation succeeds. 2035–2045: multimodal embryo-selection support may become feasible if it demonstrates added value over existing approaches.	Prospective studies linked to implantation, pregnancy, and live-birth outcomes; external validation; comparison with expert embryologists and existing grading systems; predefined evidence of clinical benefit.	High-quality annotated datasets; standardized imaging; regulatory acceptance; integration with IVF laboratory workflows; human oversight.	High
Smart cryobanking systems	Digital inventory, automated tracking, and monitoring technologies are increasingly feasible, although predictive models for long-term cryostorage outcomes remain less mature.	2026–2030: automated tracking and digital inventory. 2030–2035: predictive models for postthaw outcomes if adequately validated. 2035–2045: adaptive cryobiological management may emerge if predictive performance is demonstrated.	Validation against actual postthaw viability and recovery outcomes; reduction in identification or handling errors; demonstrated reliability of environmental monitoring; predefined prediction-performance thresholds.	Sensor reliability; data integration; interoperable databases; cybersecurity; laboratory accreditation and regulatory requirements.	Moderate
Organ-on-chip systems	Reproductive organ-on-chip and microphysiological models remain predominantly experimental, with translational relevance and standardization still developing.	2026–2030: continued experimental validation. 2030–2035: possible translational or limited clinical integration if reproducibility and manufacturing barriers are resolved. 2035–2045: multiorgan reproductive platforms may become feasible under favorable validation and regulatory conditions.	Reproducibility across laboratories; physiological validity; correlation with established biological or clinical outcomes; standardized manufacturing; demonstration of predictive value over existing experimental models.	Standardized fabrication; scalable manufacturing; validated biological endpoints; regulatory qualification; quality-control standards.	High
Digital reproductive twins	Patient-specific computational modeling is an emerging research area; clinically validated reproductive digital twins are not yet established.	2026–2030: research-stage computational modeling. 2030–2035: possible personalized fertility simulations if prospective predictive accuracy is demonstrated. 2035–2045: decision-support applications may emerge if clinical benefit is established.	Prospective prediction of clinically relevant reproductive outcomes; external validation; calibration across populations; demonstrated improvement over conventional clinical prediction methods.	Multimodal clinical, imaging, genomic, and longitudinal data; interoperability; privacy protection; validated computational models; regulatory oversight.	Very high
Multiomics fertility profiling	Genomic, proteomic, metabolomic, and other omics approaches show potential for reproductive biomarker discovery, but clinical utility, reproducibility, and standardization remain variable.	2026–2030: validation of candidate biomarkers and standardized assays in independent cohorts; 2030–2035: potential translational or selective clinical implementation if reproducible associations and clinical utility are demonstrated; 2035–2045: integrated systems-level modeling may become feasible if sustained predictive value is demonstrated.	Independent validation of candidate biomarkers; prospective outcome prediction; assay standardization; reproducibility; clinically meaningful improvement in diagnosis or treatment selection; cost-effectiveness.	Standardized assays; high-quality longitudinal datasets; bioinformatics infrastructure; data interoperability; regulatory qualification.	High
Epigenetic quality-control systems	Candidate reproductive epigenetic biomarkers are being investigated, but clinical validation and causal interpretation remain limited.	2026–2030: identification and preliminary validation of candidate biomarkers. 2030–2035: possible clinical monitoring if regulatory-grade validation is achieved. 2035–2045: integrated epigenetic risk assessment may become feasible if associations with meaningful outcomes are confirmed.	Replication across independent cohorts; standardized assays; longitudinal validation; demonstrated association with clinically relevant outcomes; predefined analytical-performance criteria.	Assay standardization; interpretation of biological variability; regulatory validation; longitudinal datasets; clinical guidelines.	Very high
Robotic reproductive laboratories	Semiautomation and robotics are increasingly feasible for selected laboratory procedures, but fully autonomous reproductive laboratories remain limited by safety and oversight requirements.	2026–2030: semiautomated workflows. 2030–2035: AI-assisted laboratory optimization if safety and error-rate benefits are demonstrated. 2035–2045: selective autonomous functions may emerge under appropriate human oversight.	Demonstrated reduction in procedural errors and variability; maintenance or improvement of laboratory outcomes; reliability testing; failure-mode assessment; predefined human-intervention thresholds.	Robotics hardware; validated software; cybersecurity; accreditation; regulatory approval; human-oversight frameworks.	Moderate–high
Global reproductive governance frameworks	Regulatory and ethical frameworks already exist but vary substantially across jurisdictions and technologies.	2026–2030: development and refinement of national and regional frameworks. 2030–2035: possible expansion of international harmonization. 2035–2045: integrated international oversight could develop if sustained consensus is achieved.	Adoption of interoperable regulatory principles; transparent ethical standards; mechanisms for cross-border governance; demonstrated consistency in implementation.	Political agreement; jurisdictional differences; ethical consensus; international coordination; enforceable standards.	Very high
Space reproductive biotechnology	Evidence remains primarily experimental, with major uncertainties concerning microgravity, radiation, gametogenesis, endocrine regulation, and embryonic development.	2026–2030: foundational experimental research. 2030–2035: prototype reproductive-support systems may be investigated if foundational evidence is favorable. 2035–2045: specialized systems could be explored for long-duration missions only if preceding research establishes sufficient safety and feasibility.	Reproducible experimental evidence; validated biological safety measures; demonstration of reproductive/developmental function under relevant environmental conditions; predefined mission-safety criteria.	Long-duration spaceflight infrastructure; radiation protection; life-support systems; ethical and regulatory approval; substantial research funding.	Extremely high

**Note.** Projected timelines represent conditional scenarios rather than predictions of inevitable technological progression. Advancement from one stage to another requires satisfactory evidence of technical feasibility, reproducibility, safety, clinical or operational benefit, regulatory acceptability, interoperability, and economic feasibility. Technologies may progress at different rates, remain investigational, or fail to advance if predefined validation or success criteria are not met.

## Data Availability

No new data were created or analyzed in this study. Data sharing is not applicable to this article.

## Abbreviations

The following abbreviations are used in this manuscript:

AI	Artificial Intelligence
ART	Assisted Reproductive Technology
CASA	Computer-Assisted Sperm Analysis
CNN	Convolutional Neural Network
DOHaD	Developmental Origins of Health and Disease
DNA	Deoxyribonucleic Acid
ICSI	Intracytoplasmic Sperm Injection
IoT	Internet of Things
IVF	*In Vitro* Fertilization
ML	Machine Learning
MPS	Microphysiological System
OOC	Organ-on-a-Chip
RFID	Radio Frequency Identification
RNA	Ribonucleic Acid
ROS	Reactive Oxygen Species
SCD	Sperm Chromatin Dispersion
SCSA	Sperm Chromatin Structure Assay
SDF	Sperm DNA Fragmentation
TUNEL	Terminal Deoxynucleotidyl Transferase dUTP Nick End Labeling
